# Browsing herbivores improve the state and functioning of savannas: A model assessment of alternative land‐use strategies

**DOI:** 10.1002/ece3.8715

**Published:** 2022-03-18

**Authors:** Katja Irob, Niels Blaum, Selina Baldauf, Leon Kerger, Ben Strohbach, Angelina Kanduvarisa, Dirk Lohmann, Britta Tietjen

**Affiliations:** ^1^ Freie Universität Berlin Theoretical Ecology Institute of Biology Berlin Germany; ^2^ Plant Ecology and Nature Conservation University of Potsdam Potsdam Germany; ^3^ 59197 Agriculture and Natural Resources Sciences Namibia University of Science and Technology Windhoek Namibia; ^4^ Berlin‐Brandenburg Institute of Advanced Biodiversity Research (BBIB) Berlin Germany

**Keywords:** browsing, ecohydrology, land use, plant community, savanna, wildlife management

## Abstract

Changing climatic conditions and unsustainable land use are major threats to savannas worldwide. Historically, many African savannas were used intensively for livestock grazing, which contributed to widespread patterns of bush encroachment across savanna systems. To reverse bush encroachment, it has been proposed to change the cattle‐dominated land use to one dominated by comparatively specialized browsers and usually native herbivores. However, the consequences for ecosystem properties and processes remain largely unclear. We used the ecohydrological, spatially explicit model EcoHyD to assess the impacts of two contrasting, herbivore land‐use strategies on a Namibian savanna: grazer‐ versus browser‐dominated herbivore communities. We varied the densities of grazers and browsers and determined the resulting composition and diversity of the plant community, total vegetation cover, soil moisture, and water use by plants. Our results showed that plant types that are less palatable to herbivores were best adapted to grazing or browsing animals in all simulated densities. Also, plant types that had a competitive advantage under limited water availability were among the dominant ones irrespective of land‐use scenario. Overall, the results were in line with our expectations: under high grazer densities, we found heavy bush encroachment and the loss of the perennial grass matrix. Importantly, regardless of the density of browsers, grass cover and plant functional diversity were significantly higher in browsing scenarios. Browsing herbivores increased grass cover, and the higher total cover in turn improved water uptake by plants overall. We concluded that, in contrast to grazing‐dominated land‐use strategies, land‐use strategies dominated by browsing herbivores, even at high herbivore densities, sustain diverse vegetation communities with high cover of perennial grasses, resulting in lower erosion risk and bolstering ecosystem services.

## Graphical abstract

We used an ecohydrological savanna model to thoroughly analyze the contrasting effects of land use by grazing and browsing herbivores. Our results confirm the well‐known phenomenon of bush encroachment with negative impacts on biodiversity and ecological processes under heavy grazing. Our highlight is that browser‐dominated communities did not lead to ecosystem degradation but to higher provision of ecosystem services, even at high densities.

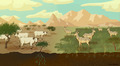

## 1 INTRODUCTION

Savannas are woodland–grassland ecosystems that provide multiple ecosystem services, including the provision of food and clean water, sequestration of carbon, preservation of biodiversity, and recreational opportunities (Sala et al., 2017). Due to highly variable rainfall and low water availability in semi‐arid savannas, agriculture is not feasible; therefore, commercial livestock are a major source of income (Adeel et al., 2005). Extensive livestock production offers some flexibility to adapt to demanding and variable environmental conditions and provides household security in drought years (Barrett, 1992; Olbrich et al., 2012). Nevertheless, farmers must balance economic sustainability and ecological capacity of a rangeland (Smet & Ward, 2005), and unsustainable land use has caused severe and widespread degradation. A major pattern in livestock‐dominated savannas (especially those where fire is suppressed) is bush encroachment, the undesirable change in vegetation community composition from perennial grasses to woody shrubs and trees (Zeidler et al., 2002). Bush encroachment is thought to result from cattle's preferential feeding on grasses and other herbaceous plants, which releases woody species from resource competition. Additionally, grazing livestock can benefit woody plants by dispersing their seeds and by removing the fuel load, thereby reducing fire frequency and intensity, enhancing woody plant recruitment (Briske, 2017). This shift toward shrubland is usually accompanied by a shift from perennial to annual grasses (Schlesinger & Jasechko, 2014; Seely & Jacobson, 1994) or from palatable to less palatable species (Milton, 1994).

The bush encroachment within savanna systems also implies changes in important ecosystem functions. Reduced cover of herbaceous vegetation leads to increased water losses and consequently accelerated soil erosion (Archer et al., 2017), as surface water runoff is enhanced due to the lower surface roughness and reduced infiltration in such areas (Tietjen et al., 2009). In general, the water cycle can be strongly altered, as woody species extract water from deeper layers (Case et al., 2020) and at the same time alter infiltration, soil water storage, transpiration, interception, and subsurface pathways that affect groundwater recharge (Acharya et al., 2018). The resultant reduced water availability puts further pressure on the performance of grasses and leads to decreases in forage quality and productivity per unit area (Egoh et al., 2012; Schlesinger & Jasechko, 2014).

Furthermore, climate change is projected to exacerbate the impact of unsustainable land use and is likely to exert a strong impact on ecosystem functions of savannas (Criado et al., 2020; Hoffmann et al., 2002; Jackson et al., 2002; Reid, 2007). The projected co‐occurrence of more frequent and intense climatic extremes (i.e., droughts) and warmer climate (Shukla et al., 2019) with heavy grazing might lead to a decrease in overall rangeland productivity and biodiversity to an extent that is economically unsustainable and cannot be ecologically reversed without interventions (Abraham et al., 2019; Milton, 1994; Polley et al., 2017).

Responding to widespread environmental degradation and in anticipation of future challenges imposed by global change, many southern African countries have started to shift from livestock production to integrated wildlife systems, such as game hunting or conservancies, as the use of wildlife can contribute to restore degraded rangelands, lead to recovery of (threatened) wildlife populations, and conserve biodiversity (Adeel et al., 2005; Holechek & Valdez, 2018; Langholz & Kerley, 2006; Richardson, 1998). Moreover, native wildlife potentially lead to higher yield in economic returns, given that they are better adapted to unfavorable climatic conditions and more resistant to endemic diseases than cattle (Cleaveland et al., 2009). Furthermore, wildlife does not only offer an alternative for red meat production and other consumptive uses, but its unique diversity is also attractive for tourism‐related activities (Richardson, 1998). Despite the current trend toward more wildlife‐based land management, there are still major research gaps on the effects of managed wildlife on vegetation composition, resulting plant functional diversity and related ecosystem functions, specifically compared to the impacts of cattle. In the past, large browsers have been shown to be a successful management tool, especially when combined with other management measures, such as prescribed fires, because they reduce the competitive effect of trees on grasses and affect fuel load (Holdo, 2007; Holdo et al., 2009; Langevelde et al., 2003). Compared to domestic cattle, native herbivore communities include a broad range of animal species of different sizes and feeding preferences. Their ability to feed on various parts of herbaceous and woody plant species can result in an overall more flexible and also balanced use of primary production and might have important implications for vegetation cover and its plant functional diversity as well as resulting feedbacks with ecosystem functions and services. However, the long‐term effects of rewilding on savanna plant communities, and in particular the role of browsing herbivores, remain unclear (Butt & Turner, 2012; Gitau, 2009; Richardson, 1998). Hence, the question arises whether native herbivores that have co‐evolved with savanna vegetation and that feature a variety of feeding behaviors can alter the composition and abundance of plant communities, which could also potentially have positive impacts on ecosystem functioning (Holechek & Valdez, 2018; Polley et al., 2017; Volder et al., 2010).

With this study, we aimed to compare impacts of two different herbivory types (grazing and browsing animals) on total plant cover, functional composition and diversity, and selected ecosystem functions. For this, we evaluated both extreme ends of the grazing–browsing gradient within herbivore communities to span the widest possible range, but also allowed for some intake of the other vegetation type to account for some flexibility in herbivory diet under unfavorable conditions, when the preferred fodder type is limited.

We parameterized our simulation model using a Namibian savanna. This question is particularly relevant to Namibia because transitions from livestock to wildlife are being incentivized by governmental initiatives (Government of the Republic of Namibia, 2014; Jones, 2015; Lindsey et al., 2013).

Modeling land use by grazing livestock and its effects in drylands is a very common practice (Kiker & Scogings, 2019; Koomen & Borsboom‐van Beurden, 2011), but only very few studies included also the removal of woody vegetation by browsers (Holdo et al., 2009; Knegt et al., 2008; Langevelde et al., 2003).

So far, no modeling study has attempted to directly compare the effects of land use by grazer‐ with land use by browser‐dominated animal communities on plant functional composition and related ecosystem processes and properties, especially related to soil hydrology. Another limitation of many of these studies is that they only included broad vegetation categories, so called plant functional types based on broad plant functional groups (PFTs), without considering the great diversity within these groups (e.g., by implementing distinguishable subtypes) (Pappas et al., 2016). However, ecosystem responses to altered drivers cannot necessarily be adequately captured when plants are grouped into very broad types, without recognizing that plant communities respond to local environmental conditions by adapting their traits, as existing simulation studies on ecosystem responses toward grazing could show (Guo et al., 2016; Lohmann et al., 2018).

Here, we built on the approach of the aforementioned studies and use the ecohydrological model EcoHyD (Guo et al., 2016; Lohmann et al., 2012; Tietjen et al., 2009, 2010) to compare the effects of grazing and browsing herbivores on the plant community composition of a savanna system and on selected resulting ecosystem properties and functions. For this, we applied a range of grazing and browsing scenarios (either dominated by grazing or browsing herbivores) to a Namibian rangeland that has been transformed from a livestock farm to a private game reserve. Our grazing scenarios allowed almost no browsing, whereas feeding in the browsing scenario occurs more selective but with a strong preference on woody vegetation. We implemented several subtypes of three broad PFTs, namely shrubs, perennial grass vegetation, and annual grass vegetation, which followed different plant life strategies (in the following called strategy types) that are characterized by specific adaptations to environmental conditions and their trade‐off.

In particular, we addressed the following three questions: (i) How does plant functional abundance and composition respond to different land‐use types and intensities and which plant life strategies prevail under specific herbivore pressure? (ii) How does this affect species richness and species evenness in the community? (iii) Do these changes affect water‐related properties, in particular water availability, in the soil and water use by plants?

We hypothesized (i) that this approach would reproduce the well‐known phenomenon of bush encroachment, (ii) that browser‐dominated communities would maintain a balance between trees and grasses due to the reduced herbivory pressure on grasses, and (iii) that browsers would positively affect biodiversity and (iv) improve water uptake by plants, altogether supporting higher grass cover.

Altogether, this study illustrates the most sustainable land management strategies to avoid soil degradation and preserve crucial ecosystem services in the long term.

## 2 METHODS

### 2.1 Study area

The private wildlife reserve Etosha Heights in Namibia (S19.1554° E15.1705°E) was established in 2002 when the transformation of eight cattle farms took place, opening the farm boundaries and merging the farms into one entity, which is now the Etosha Heights Private Reserve.

Since then, Etosha Heights was used as a game hunting lodge until 2016 when the management was changed to tourism activities and conservation research. It is now one of the largest private game reserves that offers robust conservation of wildlife (Nortjé, 2019).

Etosha Heights covers an area of 48,000 ha on the south‐western border of Etosha National Park for about 68 km in distance.

The reserve lies at 1195 m and varies between hilly patches and open plains. The site selected for this study shows little variation in topography. Precipitation is highly variable and occurs mainly during the summer months from October to April. We retrieved mean climate data from a local station and could determine a mean annual precipitation (MAP) of 298.67 ± 30.20 mm and a mean annual temperature (MAT) of 26°C, with its lowest value in June (mean of 19°C) and its highest in October (mean of 29°C). Most of the soils at Etosha Heights can be classified as “Coega” soils or Petric calcisols (Nortjé, 2019). The soil type of our study site was loamy sand.

Etosha Heights shows a very diverse landscape with many different habitats. The eastern part is patched with hills covered by mopane trees (*Colophospermum mopane*), general mopane‐veld, and open plains. The central part contains a hilly mountain range covered by a mixed habitat which is dominated by *Terminalia*, *Combretum*, and *Commiphora* species. In the west, the habitats consist of patches of mopane and acacia species.

### 2.2 Model description

We used the ecohydrological, spatially explicit dryland model EcoHyD, which is based on Tietjen et al. (2009, 2010) and was extended by a component for cattle grazing by Lohmann et al. (2012) and by a higher resolved description of plant diversity by Guo et al. (2016) and Lohmann et al. (2018). We simulated a total area of 2.25 ha consisting of 30 × 30 grid cells, with a 5 × 5 m^2^ resolution. The model has been applied and validated to several dryland ecosystems in previous studies (Lohmann et al., 2012; Tietjen et al., 2009).

The model consists of two dynamically linked process‐based submodels: a vegetation submodel and a hydrological submodel. These submodels and selected processes relevant to this study are briefly described in the following, while a comprehensive model description and model parameters can be found in Appendix S1.

The vegetation submodel simulates the fate of various functional types (strategy types) that belong to one of three broad PFTs (meta‐PFTs), namely shrubs, perennial grass vegetation, and annual grass vegetation. These strategy types differ in their traits, but follow the same processes defined by their respective meta‐PFT. The fate of each strategy type is calculated with a biweekly resolution during the wet season, which starts and ends with the first and last rain event of 5 mm/day or more during summer. Vegetation dynamics are driven by processes such as growth, mortality, competition for water and space, dispersal and establishment of seeds and seedlings, and herbivory by grazing and browsing herbivores (see section *Grazing and browsing herbivory*). Although we acknowledge that fires can also be an important driver of savanna dynamics, we did not consider fires in this analysis, as they do not occur frequently at our study site and are usually suppressed. Plant dynamics are directly linked to soil water dynamics: growth is linked to transpiration; although dispersal is not directly linked to water, dispersed seeds can only translate into new cover given sufficient water availability; and plant mortality is caused by water scarcity or senescence and is updated at the end of a fixed growing season, which runs from October to April. For grass, we assume spatially uniform dispersal within a simulated area of 150 m^2^, while shrub dispersal is restricted to a local dispersal kernel with individual larger dispersal events being facilitated by large herbivores.

The hydrological submodel simulates daily dynamics of surface water and soil moisture in two soil layers. Water dynamics are driven by precipitation, lateral water redistribution of surface water, infiltration, vertical fluxes, and water losses via evaporation and transpiration. Infiltration of surface water into the soil is linked to vegetation cover, as we assumed a positive effect of plant roots on soil porosity. Runoff decreases with increasing vegetation cover, first because of the higher infiltration facilitated by plant roots, and second because of a higher surface roughness delaying the water flow. Water losses are linked to plant root proportion in the two soil layers and to vegetation cover, which decreases evaporation but increases water use by plants and therefore transpiration. Annual grasses only have access to water in the upper soil layer, while perennial grasses and shrubs have access to water in both layers according to their root distribution (Case et al., 2020).

### 2.3 Model input

The model was parameterized to the environmental conditions of Etosha Heights Private Reserve, specifically topography and soil, climatic conditions, and plant species characteristics.

#### 2.3.1 Topography and soil

We added a digital elevation map (DEM) corresponding to the position of the weather station to account for local differences in topography. We retrieved the DEM from the Shuttle Radar Topography Mission (SRTM) (EROS Center, 2017) and linearly interpolated it from a resolution of 30 × 30 m^2^ to 5 × 5 m^2^.

We parameterized the soil based on the standard data set of EcoHyD for loamy sandy soils (Rawls et al., 1992). We additionally calibrated selected soil parameters of the hydrological submodel fitted to a 1.5‐year time series of soil moisture recorded at Etosha Heights (Figure S3.1) using the corresponding data on temperature and precipitation of the weather station (coordinates of weather station: S19.245323◦ E15.191395◦). The station measured soil moisture at 5‐minute intervals using sensors (SM150T, Dynamax, UK) in three depths (10, 50, 100 cm) under woody cover and in open plain. These soil parameters included residual water content and wilting points of soil and plants, as well as the evaporation constant accounting for reduced evaporative losses in the presence of soil crusts. We set vertical fluxes by diffusion to zero, as the measured data suggest that pore size is too high to allow for capillary transportation by suction (Nimmo, 2013). Furthermore, as the measured soil moisture data showed almost no response of soil moisture in the deeper soil to precipitation input and remained rather constant over time, we only allowed very little diffusion from the upper to the lower soil layer. Parameter values were chosen by determining the lowest root mean square error (RSME) when comparing measured and simulated soil moisture data after a local sensitivity analysis (RSME = 1.092).

#### 2.3.2 Precipitation and temperature of the simulation experiment

As vegetation dynamics can be sensitive to rainfall conditions and savannas are exposed to highly variable precipitation events, we approached the resulting uncertainty by replicating simulation runs with artificial stochastic precipitation time series. We generated stochastic precipitation time series using the precipitation generator NamRain as described by Tietjen et al. (2010) and Lohmann et al. (2012). The time series were based on a precipitation time series from the Tropical Rainfall Measuring Mission (TRMM) (Huffman et al., 2010) measuring precipitation at a three‐hourly rate from 1998 to 2014. We compared the time series to five‐year long rain gauge data on site and concluded that they closely match. A comparison of monthly distribution showed a correlation coefficient of 0.98 (Spearman's Rho), but rainfall peaks were underestimated to some extent. Other studies using TRMM data for precipitation measurement also found out that TRMM data are a reliable estimation, but daily rainfall values are slightly underestimated compared to rain gauge recordings (Tarek et al., 2017; Yong et al., 2015). We therefore applied a calibration factor of 1.16 to the TRMM data to reduce the deviation from station data. Temperature was fitted according to MAT, yearly, and daily fluctuations.

In total, 30 stochastic precipitation time series along with temperature with an hourly resolution were generated. The time series ranged from annual precipitation sums of 53.0 mm to 837.6 mm. The MAP of all climate repetitions was 291.3 mm, and they were highly correlated with the TRMM time series (*R* ^2^ = 0.972, Spearman's Rho = 0.986).

#### 2.3.3 Assemblage of plant strategy types

Three major PFTs are represented in the model: woody vegetation, perennial, and annual herbaceous vegetation. In this study, these so‐called meta‐PFTs “woody vegetation” (hereafter referred to as shrubs) and “perennial herbaceous vegetation” (perennials) were further divided into subtypes representing particular life strategies to understand the effects of herbivory on a functionally more diverse plant community. As annual grasses (annuals) only dominate degraded systems (Archer et al., 2017; Case et al., 2020), we did not subdivide them further. We used the standard set of parameters of the meta‐PFTs from previous studies (Guo et al., 2016; Lohmann et al., 2018) as starting point for parameterizing the subtypes. The basic subtype of each meta‐PFT (hereafter called “base type”) had the same set of parameters as the original meta‐PFT. We then derived additional subtypes (for shrubs and perennials) by defining individual life strategies that show trade‐offs in two particular characteristics, but are otherwise the same as the base type. Based on major species found at Etosha Heights (Table 1), the simulated trade‐offs in this study include characteristics related to six processes (Tables 2 and S1.1): (i) biomass production B (represented by parameter *T*_veg_; used in Equation S1.6), (ii) mortality *M* (parameter mrd_veg_; Equation S1.8), (iii) palatability P (parameter herbivorePref; Equation 3), (iv) defense *D* (parameter edibleFrac; Equation 3), (v) competitive strength for water C (parameter UptakeRate; Equation S1.4‐6), and (vi) resistance to drought R (parameter WiltingPoint; Equation S1.8). A very drought‐resistant subtype could for example show a very low biomass production as trade‐off.

**TABLE 1:** Plant life strategies of perennial grasses and shrubs and respective example species at Etosha Heights

Perennial strategy	Example species	Shrub strategy	Example species
Bd	*Stipagrostis uniplumis* *Oropetium capense*	Bd	*Grewia olukondae* *Grewia villosa*
Bp	*Stipagrostis uniplumis*	Bp	*Acacia mellifera, Terminalia prunioides, Terminalia stuhlmannii, Colophospermum mopane*
Cb	*Eragrostis nindensis* *Eragrostis trichophora* *Bothriochloa radicans*	Bc	*Acacia kirkii s. kirkii v. kirkii*
Cp	*Eragrostis echinochloidea*	Cb	*Asparagus*
Pb	*Eragrostis trichophora* *Oropetium capense*	Db	*Asparagus*
Pr	*Bothriochloa radicans*	Dc	*Acacia nebrownii*
Rb	*Eragrostis nindensis* *Eragrostis trichophora*	Dr	*Acacia kirkii s. kirkii v. kirkii*
Rp	*Stipagrostis uniplumis*	Mb	*Terminalia pruniodes*
		Rc	*Acacia nebrownii*
		Rd	*Grewia olukondae*

**TABLE 2:** Parameter settings of perennial grasses and shrubs for the cattle and wildlife scenarios. The table lists the parameter name, how it is referred to in the text and figures, its default value, and the value used for trade‐off (Parameter −10%) and specialization (Parameter +10%)

Parameter	Short	Land use by grazers			Land use by browsers		
		Default	−10%	+10%	Default	−10%	+10%
Perennials							
Defense	D/d	0.15	0.125	0.175	0.95	0.944	0.956
Palatability	P/p	1	1.030	0.970	0.2	0.898	0.000
Mortality	M/m	0.54	0.557	0.523	0.54	0.556	0.524
Biomass production	B/b	0.5	0.484	0.516	0.5	0.482	0.518
Competitive strength for water	C/c	0.9	0.879	0.921	0.9	0.876	0.924
Resistance to drought	R/r	0.077	0.0774	0.0766	0.077	0.0774	0.0766
Shrubs							
Defense	D/d	0.95	0.924	0.976	0.7	0.614	0.786
Palatability	P/p	0.2	0.243	0.157	1	1.291	0.709
Mortality	M/m	0.12	0.137	0.103	0.12	0.136	0.104
Biomass production	B/b	0.15	0.143	0.157	0.15	0.142	0.158
Competitive strength for water	C/c	0.5	0.479	0.521	0.5	0.471	0.529
Resistance to drought	R/r	0.076	0.077	0.075	0.076	0.083	0.069

In order to generate comparable trade‐offs for each strategy type, we used a sensitivity analysis, in which we separately determined for each of the six altered parameters, which parameter value led to a cover increase or decrease of 10% in subtype cover compared to simulations with the default parameter value (Figure S4.1). For this, we evaluated the subtype cover of the last 20 years of 100‐year simulation runs. The resulting parameter values were then used to parameterize strategy types with a beneficial value for one parameter and an adverse value for another parameter (Table 2).

In the following text and figures, we referred to these strategy types by their strategy name, which always consists of a capital and a lowercase letter, corresponding to the traits listed above. The first capitalized letter refers to the specialization in a specific property, that is, the trait the plant benefits from by increasing its cover by 10%. The lowercase letter represents its trade‐off, which results in a 10% cover decrease (Table 1).

#### 2.3.4 Model parameterization of plant species and sensitivity analysis

We performed a sensitivity analysis for perennial grass vegetation and shrubs to determine which parameter values of the above described strategies resulted in a 10% cover increase or decrease relative to the mean cover of the base type of the respective meta‐PFT. However, as the performance of plants is highly dependent on their interactions with other plants, we ran these simulations in presence of other plants: For each parameter related to a specialization, we have gradually changed its value in a range of up to ±30% relative to the default value of the respective base type. The performance (i.e., resulting cover) of this altered type was assessed in a simple plant community consisting of this altered type, the original base type of the same meta‐PFT (both with dynamic cover) and the base types of the two other meta‐PFTs (both with static cover). We assumed a linear relationship between the parameter value and resulting cover of the altered type. Based on this assumption, we used a linear regression analysis to determine which change in the parameter value results in a +10% or −10% deviation in cover. Only parameters with a significant slope were used for parameterization.

The 100‐year sensitivity analysis simulations were conducted separately for each meta‐PFT. To minimize random effects during the initialization process, shrubs and perennials were initialized with a fixed cover of 20%. All simulations were repeated for the thirty 100‐year time series of stochastic rainfall to account for rainfall variability. We applied a medium animal density (stocking density = 40 ha/LSU), allowing grasses and shrubs to coexist. One livestock unit is defined as a 450 kg live herbivore that ingests 2% of its body weight daily (Bothma & du Toit, 2016). We conducted the sensitivity analysis separately for the land‐use type grazer‐dominated and browser‐dominated (Table 3), as the sensitivity of model results was highly dependent on the specific herbivore pressure.

**TABLE 3:** Settings of different land‐use scenarios in terms of herbivory pressure, animal density (SR), and plant parameters of the respective default PFT influencing biomass removal. Specific plant parameters can vary depending on plant life strategy

Scenario	Land‐use type	Stocking rates [ha/LSU]	Feeding settings shrubs	Feeding settings perennials	Feeding settings annuals
Grazing (very low, low, medium, high, very high)	Grazing dominated	50, 40, 30, 20, 10	herbivorePref = 0.2 edibleFrac = 0.95	herbivorePref = 1 edibleFrac = 0.15	herbivorePref = 0.6 edibleFrac = 0.05
Browsing (very low, low, medium, high, very high)	Browsing dominated	50, 40, 30, 20, 10	herbivorePref = 1 edibleFrac = 0.7	herbivorePref = 0.2 edibleFrac = 0.95	herbivorePref = 0.1 edibleFrac = 0.95

This resulted in a total of 21 strategy types, of which 10 shrub and 8 perennial grass strategies each resulted from the analyses described above and, in addition, the base type of each meta‐PFT was also included (Tables 1 and 2). Results of the sensitivity analysis can be found in Appendix S4.

### 2.4 Grazing and browsing herbivory

The actual removal of biomass by herbivory was calculated at the end of the growing season. The herbivory algorithm can be divided into four main parts: estimation of above ground biomass (BM) from vegetation cover, calculation of the biomass demand and of edible biomass, removal of biomass by animals, and reconversion from biomass to vegetation cover.

As the model simulates vegetation cover rather than biomass, first, aboveground biomass has to be estimated from vegetation cover, calculated by multiplying cover with a conversion constant (*conv_c_bm_veg_)*. This term is then fitted to the precipitation sum of the respective year and MAP (*cf_rain_*) to predict plant growth depending on water availability (Lohmann et al., 2012).

(1) $${\text{BM}}_{\text{veg}}=C_{\text{veg}}*\text{conv}\_c\_{\text{bm}}_{\text{veg}}*{\text{cf}}_{\text{rain}}$$

Biomass demand of the animals is calculated as a function of the density of animals in the landscape, which is given by the stocking rate (SR) in hectares per livestock unit (ha/LSU). This approach allows us to directly compare the effect of grazing and browsing animals that have the same overall biomass demand rather than their specific numbers or differences in per capita fodder uptake. The total needed BM consumed by animals of a certain density each year is thus defined as:

(2) $$\text{Needed}\;\text{BM}=\text{animal}\;\text{bodyweight}\;\left(\frac{\text{kg}}{\text{LSU}}\right)*365\;\text{days}*0.02\left(\frac{1}{\text{day}}\right)*\frac{1}{\text{SR}}*\;\text{gridarea}\;\left(\text{ha}\right)$$

In this study, we varied the density of animals (SR), resulting in different total biomass (BM) requirements for fodder. The lower SR, the more animals per area and the higher the total BM requirement and herbivory pressure.

Third, the edible BM (BM_edible_) of each grid cell is calculated based on green and dry grass BM of the previous year that is still edible. The amount of edible BM is constrained by how much of a strategy type can be eaten, as specified in the properties of each strategy type (parameter edibleFrac/Defense*)*. Thereafter, a random cell is drawn, and the amount of removed BM (RBM, Equation 3) is calculated based on local edible BM availability. Since several different strategy types can occur in a grid cell, the actual amount of BM consumed by each strategy type is determined by the relative palatability of each strategy type in that cell.

(3) $${\text{RBM}}_{\text{type,veg}}=\frac{\text{herbivorePref}}{\frac{\sum_{\text{veg}}{\text{herbivorepref}}_{\text{veg}}}{\text{veg}\_\text{Nr}}}\times \left(\frac{{\text{BMedible}}_{\text{type,veg}}}{\sum_{\text{veg}}{\text{BMedible}}_{\text{type,veg}}}\right)\left[\text{dimensionless}\right]$$

If the BM demand of animals exceeds the amount of edible BM in this cell, all edible BM is removed; otherwise, BM removal happens selectively. This step is repeated until the total fodder demand is fulfilled. In the event that there is nevertheless a deficit of edible biomass, we assume that the animals are provided with enough fodder to survive, implying a constant SR throughout a simulation. However, when calculating seed dispersal, we adjust the SR in case of food deficits, which means that fewer animals disperse seeds that are translated into new cover. In the final step, the amount of remaining BM of each strategy type is converted back to cover using the inverse function of Equation 1.

Thus, depending on the relative feeding preference of animals for different functional vegetation types (parameter *herbivorePref*) and the ratio of edible to nonedible biomass of plants (parameter *edibleFrac*), we can use the same herbivory algorithm for the removal of grass and woody vegetation. Both parameters are crucial in determining how tasty or palatable a plant is and which biomass fraction of it can be eaten. Parameter variation can thus be used to simulate BM removal by grazing and browsing, but also plays a role in distinguishing between the attractiveness of strategy types within each meta‐PFT group.

### 2.5 Land‐use scenarios

We tested the effects of several land‐use strategies by applying two land‐use types, namely land use dominated by grazers, preferring grasses, versus land use dominated by browsers, preferring woody species in varying intensities. We decided to focus on browsing‐dominated communities and not on an equal proportion of mixed feeders in order to have more direct and comparable effects between grass and shrub removal. In both land‐use types, we nevertheless allowed some shrub/grass intake to allow adaptative herbivory in response to changes in food availability during very dry conditions. Furthermore, by parameterizing strategy types, we additionally accounted for selective herbivory depending on the palatability and edibility of each type. The effect of land‐use intensity was tested by varying the SR from very low to very high (Table 3).

The simulation duration for every scenario was 100 years in order to identify long‐term effects. All results are based on 30 repetitions using the previously described stochastic precipitation and temperature time series. Means were generally taken from the last 20 years of each simulation run. In this paper, we focused on the results of the low (40 ha/LSU) and high (20 ha/LSU) SRs as there was little variation in‐between. The complete set of results can be found in Appendix S5.

### 2.6 Data analysis

All results were analyzed and visualized using R (R Core Team, 2019). For the statistical analyses, we evaluated the last 20 years of the simulation, in which a steady state for vegetation cover was reached.

#### 2.6.1 Abundance and composition

To clarify the effects of different types of herbivory on abundance and composition (Question 1), total cover of all PFTs was compared by conducting a Scheirer–Ray–Hare nonparametric test for two‐way factorial design looking at the factors: land‐use type and intensity. The cover of strategy types was analyzed as a function of strategy and land use. Factor levels were ordered from highest to lowest median for the post hoc analysis.To investigate differences between strategy‐types in different land uses, the post‐hoc Dunn test was conducted. To increase statistical significance, the “bh” method (Benjamini‐Hochberg) was used to adjust the p‐value. Furthermore, we calculated effect size using the nonparametric epsilon squared method (*e* ^2^), which determines everything below 0.08 as small, between 0.08 and 0.26 as medium, and above 0.26 as large effect size (Rea & Parker, 2014).

### 2.7 Biodiversity

To test the effect of herbivory on biodiversity, two biodiversity indices were calculated: richness of strategy types, counting the total number of strategy types present, and evenness (Pilou's *J*), which additionally accounts for the individual abundance and distribution of strategies. It is calculated by comparing the Shannon diversity (H) to the maximum possible diversity value (richness S):

(4) $$J=\frac{H}{\text{ln}\;S}$$

(5) $$\text{and}\;H=‐\sum_{i=1}^{k}p_{i}\;\text{log}\left(p_{i}\right)$$

with *k* denoting number of groups and $p_{i}$ denoting the proportion in group *k*. As plants are not represented as individuals, both measures were calculated using total cover in the whole simulated area as a proxy. Only strategy types with a total cover greater than 2.5% were included in the species richness calculation to remove residue or dead biomass of already near‐to‐extinct species.

We applied generalized linear mixed‐effect models (GLMMs) to richness data using the R package “nlme” (Pinheira et al., 2021) to determine the response of richness to land use. We included climate repetition as a random effect to account for non‐equal variances and to allow each repetition to have its own mean value. Richness is derived from count data, which is why we chose a Poisson distribution. To test the significance of random effects, we conducted a likelihood ratio test and compared the goodness of fit.

Evenness between land‐use types was compared by conducting a nonparametric Kruskal–Wallis rank sum test and the post hoc Dunn test, including the “bh” p‐value adjustment. Again, we calculated effect size using the nonparametric *e* ^2^ method.

#### 2.7.1 Exploratory factor analysis

Furthermore, we conducted an exploratory factor analysis (EFA) to determine the clustering of strategy types based on factors for each scenario and to explore the underlying theoretical structural relationship between strategy types and degree of cover. Based on the factorization, a cluster analysis was conducted. We calculated within‐cluster variance using the within‐cluster sum of squares (WCSS).

Additionally, factorization allowed to reduce the dimensionality of the data and the clear visualization of functional dispersion (FDis). FDis served as an estimator of the dispersion in trait combination abundances by calculating the mean distance of all strategy types to the weighted centroid of the community in the multivariate trait space (Laliberté & Legendre, 2010). Each distance between species and centroid was weighted by the relative abundance of the respective species. Since within a functional plant group all cover differences were driven solely by trait differences, the total cover was used as the basis for determining the factor structure.

Latent factors were determined by choosing only factors with eigenvalues greater than 1 and those which were above threshold in the scree plot visual test. For the simplicity of presentation and because there was sufficient explanatory power, only two of three potential factors were extracted. The two‐factor model was fitted and factor loads were determined for all PFTs. Based on the factors, two clusters could be identified which corresponded to the meta‐PFTs. We plotted the strategy type loadings along the two‐factor axes and combined this with FDis results by including the centroid and the distance axes to each strategy type representing FDis.

#### 2.7.2 Water use and availability

In order to analyze the effects of different land uses on water fluxes, we first defined relative water use by plants as the ratio of transpired water to total water losses by evapotranspiration (T/ET). Since soil water was lost rapidly in our study area, we used T/ET to analyze the productive part of the water cycle in the plant. The water fluxes regarded in this study are all green water according to the classification by Falkenmark (1995). Green water refers to the precipitation input that is stored in the soil until part of it is consumed by plant transpiration which is directly related to their biomass production. T/ET is an important factor to investigate the relationship between vegetation growth and ecosystem water use that has gained increasing recognition in recent years (Fatichi & Pappas, 2017; Schlesinger & Jasechko, 2014; Wei et al., 2017).

We checked assumptions and performed a linear regression analysis and ANOVA tests to test differences between scenarios. Furthermore, we calculated the magnitude of the effect of land use on T/ET using Cohen's *d* (Cohen, 1988).

Second, for the comparison of soil moisture, we used mean moisture during the variable wet season, which we defined as the time between the first and last day with a rainfall of more than 5 mm during that respective season. Due to heteroscedasticity of the data, a one‐way ANOVA with Welch correction was conducted.

## 3 RESULTS

### 3.1 Plant composition

#### 3.1.1 Abundance of meta‐PFTs

**FIGURE 1:**
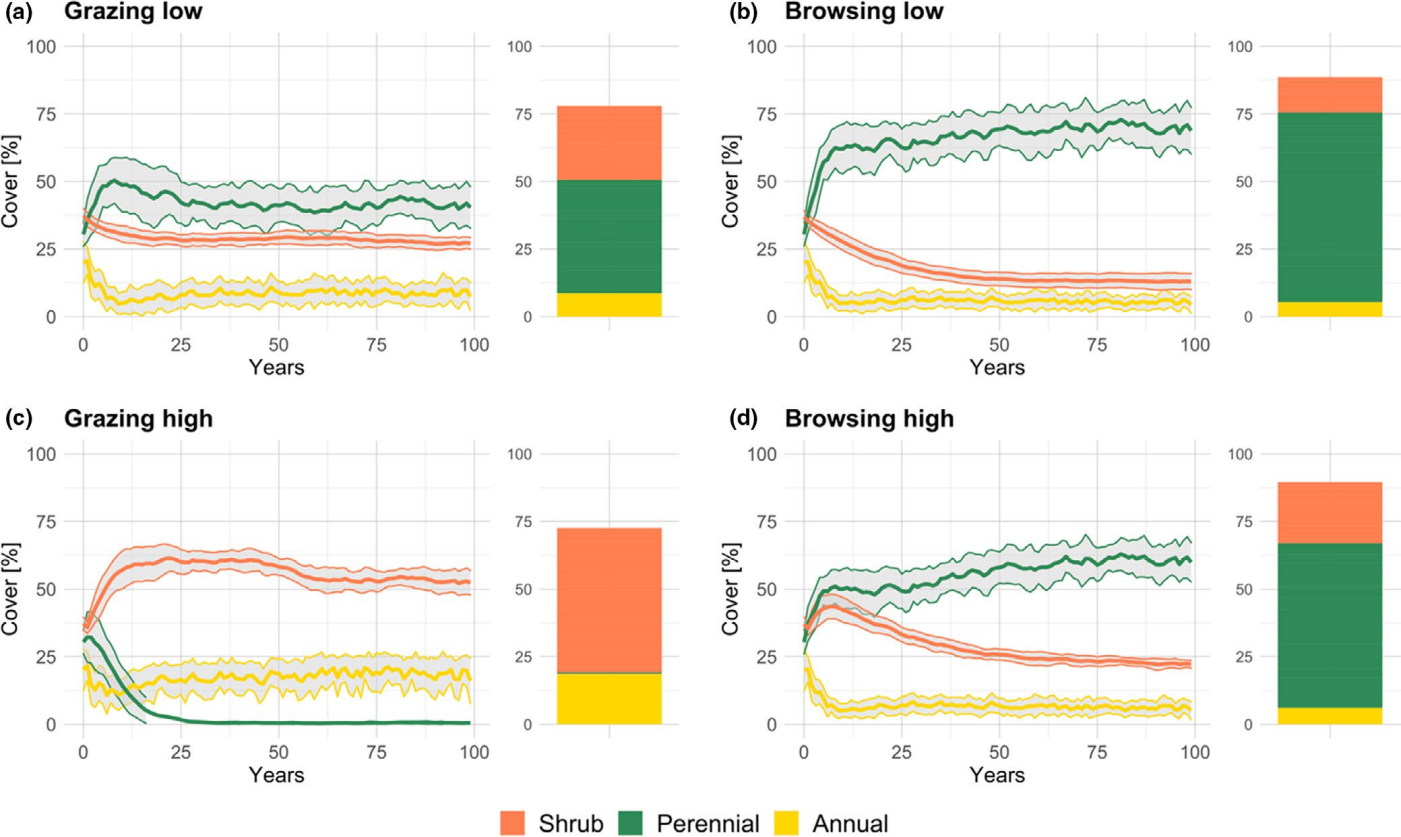
Predicted mean cover ± SD [%] for 30 climate repetitions of the three meta‐PFTs for the whole simulation duration (lines) and the last 20 years of simulation (stacked bars) for all land‐use scenarios

Land‐use type, intensity, as well as their interaction had a significant effect on total vegetation cover (Figure 1, *H* = 153.5, *p* < .001). Total cover differed between land‐use scenarios (*p* < .001) with highest total cover for the browser scenario of high intensity (Browsing high). A power analysis revealed that land‐use type (grazing vs. browsing) and intensity had a strong effect on perennial cover and thus on total vegetation cover (*e* ^2^ = 0.48).

All scenarios apart from those with a high grazer density (Grazing high, Figure 1c) resulted in a stable state where all three PFTs can coexist. Perennial grasses could not prevail under high grazing pressure as seen for high and very high grazer densities (Figure S5.1).

The collapse of the perennial grass matrix resulted in an immediate increase of shrub cover and bush encroachment and allowed annual grasses to become more abundant. Shrubs were generally more dominant in grazer scenarios (Figure 1a, c) where they did not seem to be adversely affected by herbivory. Also, shrubs benefited from high SRs as there were more animals dispersing their seeds which was in turn translated into new cover. The sparse cover of shrubs in the browser scenarios (Figure 1b, d) suggested that browsing led to an increase in perennial grass cover and therefore reduced shrub cover.

#### 3.1.2 Abundance of perennial grasses

**FIGURE 2:**
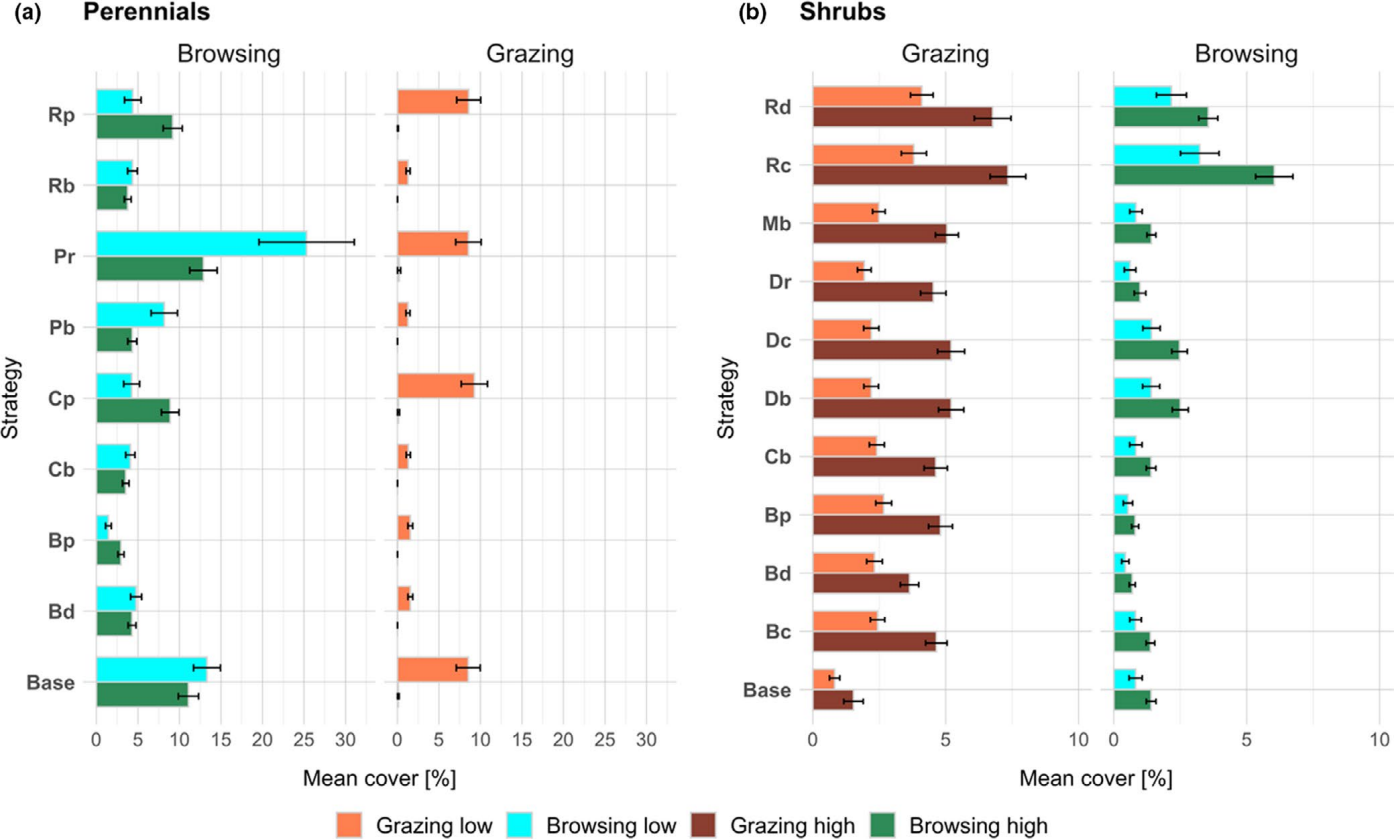
Mean cover ± SD [%] of perennial (*N* = *9*) and shrub (*N* = *11*) strategy types in different grazing and browsing scenarios, suggesting which strategy types dominate in a certain land‐use scenario. Results represent vegetation cover of the last 20 years of simulation repeated for 30 climate time series. Note that x‐ and y‐axes between perennial grasses and shrubs differ

Dominance of perennial grass species depended on herbivory type, intensity, and plant strategy (Figure 2a). Strategy and land‐use played a significant role in determining the performance of a strategy type (*H* = 19778.7, *p* < .001). The scenarios with low grazer density (Grazing low) and high browser density (Browsing high) both led to similar abundances concerning successful strategies: The predominant plant types were the base‐type, the highly water‐competitive or drought‐resistant plants with high palatability (Cp and Rp), but also the complementary strategy‐type with low palatability and low drought resistance (Pr). Under lower browsing intensity (Browsing low), the latter strategy as well as the default type became even more successful, while the cover of the other strategies decreased.

#### 3.1.3 Abundance of shrubs

Under grazing pressure, the cover of shrubs did not differ strongly between strategy types, suggesting that no specific strategy led to a higher abundance (Figure 2b). When perennial grasses increased under low cattle pressure, the amount of each shrub was reduced to the same extent.

Nevertheless, the strategies related to high drought resistance (Rd and Rc) were slightly higher in their cover as their adaptation to water stress allowed them to survive longer under very dry conditions. The only strategy type that seemed to have a disadvantage was the generalist base type, which did not follow specific adaptations or trade‐offs.

Under browsing pressure, the variation in cover between shrub strategy types was higher than under grazing. Here, Rd and Rc became the dominant types, and the strategy types Bp and Bd were drastically reduced in their cover, indicating that either the increased BM production and/or the coupled trade‐off that increased herbivory sensitivity was not advantageous under high browsing pressure.

Also, the strategies with higher defense mechanisms, Db and Dc, had an advantage under high browsing as their defense mechanism reduced the amount of edible BM (Figure 2).

### 3.2 Biodiversity

**FIGURE 3:**
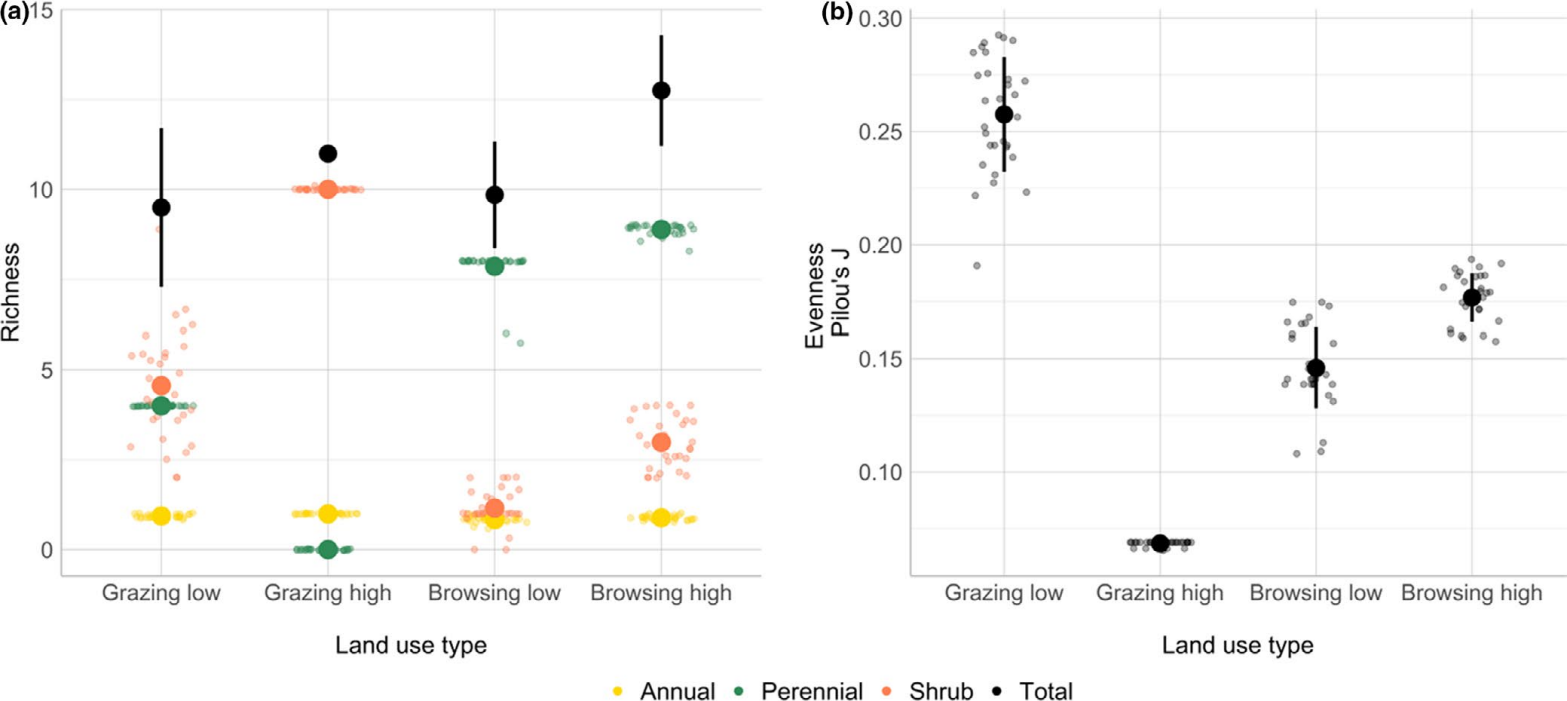
Mean ± SD of richness (a) and evenness (b) under all land‐use scenarios for 30 climate repetitions. Every point represents one climate repetition. The left figure shows the total richness of all PFTs, as well as richness of each meta‐PFT for every scenario respectively

Total richness varied slightly between scenarios, ranging from 9.49 ± 2.21 species under low grazing intensity (Grazing low) to 12.75 ± 1.54 under high browsing intensity (Browsing high). Land use in high intensity did either favor woody (grazer) or perennial grass species (browser) and led to high richness values. Both land use and PFT had a significant effect on total richness (GLMM, *p* < .001). Richness of each of the three meta‐PFT indicated that perennial grass abundance was significantly higher in browsing scenarios and decreased in scenarios with decreasing grazing pressure (Figure 3, left). Browser‐dominated land use could thus increase grass richness by a factor of 3.13 compared to grazing scenarios (GLMM, *p* < .001). Climate repetition as a random factor contributed significantly to the variation in richness (GLMM, Chi^2^ = 55.809, *p* < .001), illustrating that land‐use effects cannot always be separated from climate effects.

Evenness (Chi^2^ = 109.52, *p* < .001) varied significantly between scenarios with a very strong effect of land use on evenness (*e* ^2^ = 0.92). As nearly all species surviving under high grazing belonged to woody vegetation, resulting evenness was lowest. Browsers increased evenness, but it was still significantly lower than in the scenario with low grazing pressure, which can probably be attributed to the single dominance of strategy types and the drastic reduction in shrub cover. Evenness was highest for low grazing due to the relatively homogeneous distribution of plants and relatively high richness of species (Figure 3a, b).

#### 3.2.1 Implications of herbivory for plant diversity

**FIGURE 4:**
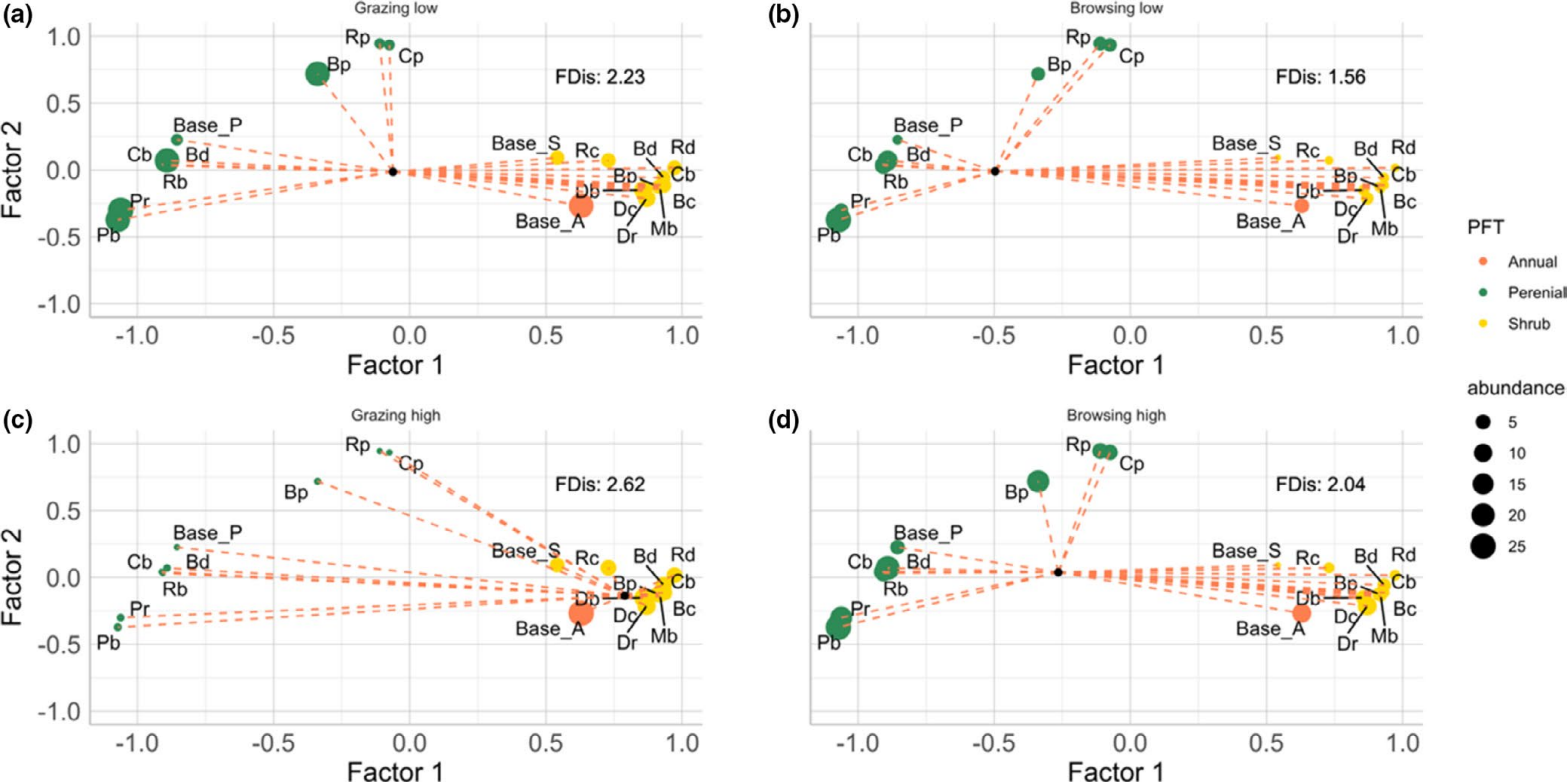
The relationship between factors correlating the occurrence of PFTs and functional dispersion (FDis). The *x*‐ and *y*‐axes denote the two factors separating the distribution of strategy types. PFTs can be distinguished by color and the points are scaled by abundance. The black point depicts the centroid, representing the weighted centroid of trait combinations in the community, and the dashed lines the distance of the respective strategy type to the centroid, weighted by its relative abundance. The clusters suggest which species are similar and thus usually occur together

Based on the factor loads, two clusters could be identified which separate the meta‐PFTs (Figure 4). Generally, when plants in Cluster One dominated Cluster Two, vegetation abundance in Cluster Two generally declined, while conditions that favored Cluster Two vegetation appeared to negatively affect vegetation in Cluster One. Plant functional trait composition responded to herbivory intensity in both community means and dispersion.

Factor one (*x*‐axis) clearly separated shrubs from grasses while the second factor (*y*‐axis) differentiated plants with high herbivory sensitivity versus plants with low herbivory sensitivity. Positive values of factor one included shrubs and annuals, and negative values included perennial grasses. Factor two included species with high sensitivity to herbivory at positive values and those not so sensitive at negative values. Thus, non‐palatable or species with high defense or high recovery mechanisms were favored under high herbivory pressure.

The species loadings were plotted along the two‐factor axes and combined with FDis results by including the centroid and the distance axes to each species representing FDis. The centroid shifted from being near shrubs under high grazing pressure (Figure 4c) toward perennial grasses in the other scenarios (Figure 4a, b, d), suggesting which meta‐PFT will dominate under which land‐use type.

The clustering of species based on total cover of all scenarios showed to which extent and in which distance to the centroid they appear in the trait space. The annual grass type was categorized as having more shrub‐like features and therefore appears in the woody cluster. These clusters indicate a smaller distance and hence fewer differences between the shrub species compared to distances between perennial grass species. The variance in the shrub cluster was therefore significantly lower (WCSS = 0.33) compared to the perennial cluster (WCSS = 3.27). Palatability seemed to be the driving factor in the variability within a cluster of perennial grasses.

Generally, FDis was maximized under high grazing pressure (FDis: 2.62), when woody vegetation dominated, while it was minimized under low browsing pressure (1.19), when the cover was overall higher and more dominated by perennial grasses, suggesting that FDis increased with disturbance.

### 3.3 Water use and availability

**FIGURE 5:**
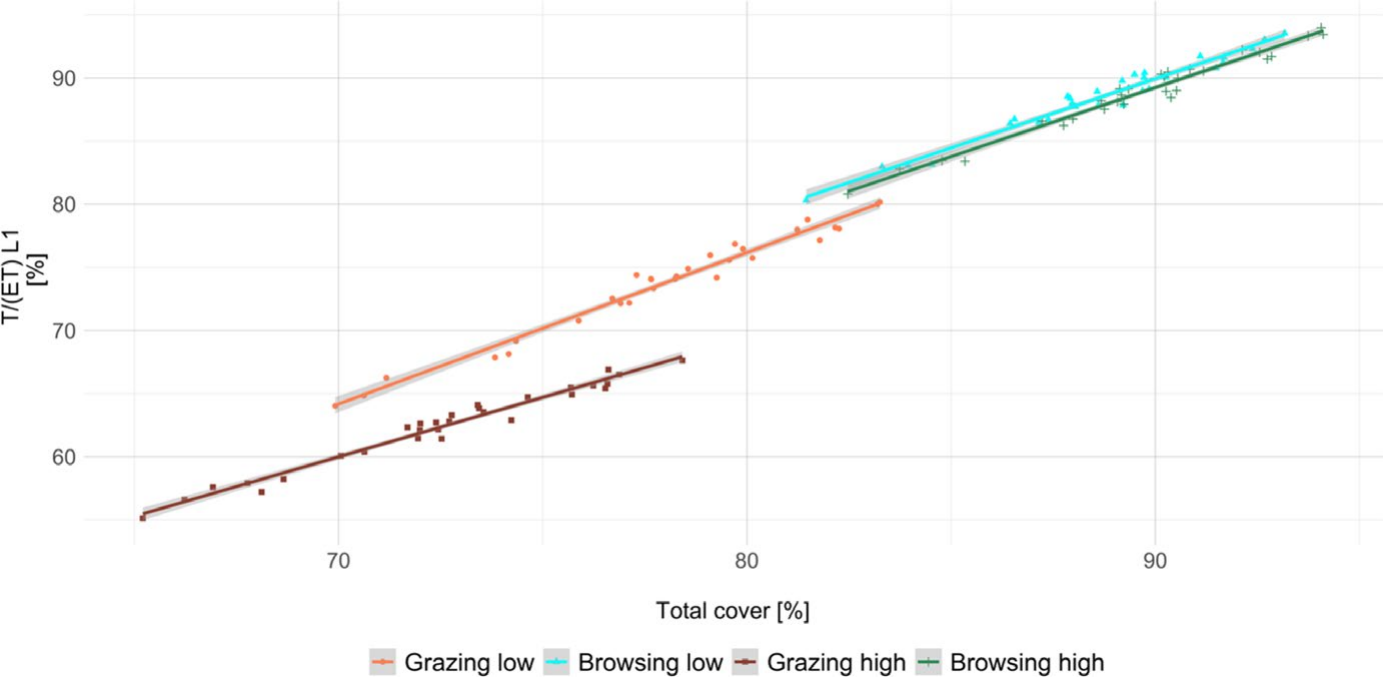
Correlation between the variation in the ratio of transpiration and evapotranspiration (T/ET) [%] as estimator for water use by plants and total plant cover [%], separated for the different scenarios of land use. Results refer to water dynamics in the upper soil layer of the last 20 years of simulation and 30 climate repetitions

For each scenario, relative water use by plants (T/ET) increased linearly with increasing total plant cover (Figure 5; *F*_7, 2392_ = 2655, *p* < .001), but cover had no effect on soil moisture (Table 4; *p* > .05). Thus, cover increased transpiration and controlled T/ET over the long term. The lowest cover coincided with the lowest water used in the high grazing scenario. Both were slightly higher in the lower grazing scenario. Remarkably, more water was available to plants in the browsing scenarios, with almost identical ranges in both, but slightly higher cover and T/ET in the low browsing scenario. In both browsing scenarios, there was almost no green water loss as almost all available water could be used for transpiration. In total, vegetation cover and land‐use scenario could explain 88.6% of the variation in water use. Cohen's *d* predicted a very large effect size of land use on relative water use (2.73).

**TABLE 4:** Mean values ± SD of total cover, relative water use by plants (T/ET), and soil moisture for each land‐use scenario

Scenario	Total cover ± SD [%]	T/ET ± SD [%]	Soil moisture ± SD [Vol %]
Grazing low	77.96 ± 7.86	73.74 ± 7.33	11.41 ± 1.74
Grazing high	72.53 ± 8.37	62.37 ± 5.54	11.34 ± 1.75
Browsing low	88.60 ± 7.21	88.41 ± 6.20	11.48 ± 1.74
Browsing high	89.64 ± 7.14	88.84 ± 6.23	11.48 ± 1.75

Although total cover and soil moisture were significantly positively correlated (*p* < .001, Spearman's Rho = 0.56), land use did not have an effect on soil moisture as soil moisture levels were similar across all scenarios (Table 4; *F*_3, 1331.1_ = 0.84, *p* > .05).

## 4 DISCUSSION

In this study, we examined potential effects of land‐use types dominated by grazers or browsers in different intensities on grass and woody PFTs composition and resulting changes in total vegetation cover, biodiversity and water availability. Our modeling approach provides a new route for studying the effects of shifting land use from grazing to browsing herbivores on plant functional diversity and the link to ecosystem functions. In the following, we address our three major questions on the response of plant abundance, composition, and diversity on herbivory by grazing versus browsing as well as on water fluxes and discuss the implications for management. We also discuss whether we could confirm our hypotheses, (i) that we were able to reproduce bush encroachment under high grazing, that browser‐dominated communities (ii) could reduce pressure on grasses and allowed a tree–grass coexistence, and (iii) had a positive effect on biodiversity and (iv) led to improved water uptake.

### 4.1 Plant composition

#### 4.1.1 Abundance of meta‐PFTs

The simulations showed that the choice of herbivores strongly affected plant community composition. High land‐use intensities drove the vegetation to be more homogeneous and dominated by either woody (grazing) or herbaceous types (browsing).

Heavy grazing led to typical degradation patterns: a decrease in total vegetation cover, a shift in the plant community toward a higher proportion of woody vegetation, and the appearance of annual vegetation. These results, also referred to as bush encroachment, are in line with findings from previous studies (Meyer et al., 2007; Nacoulma et al., 2011; Pfeiffer et al., 2019). Heavy grazing led to an increase of bare ground due to selective removal of perennial grasses. Bare ground opened a window of opportunity for the establishment of woody species, but also provided space for annual grasses (Archer et al., 2017; Polley et al., 2017; Zeidler et al., 2002).

We could also show that a decrease in grazing pressure allowed some perennial grass species to prevail, resulting in the coexistence of woody and grass species. Here, the increase of perennial grass species decreased the available space for woody vegetation and therefore their growth capacity.

In contrast, the perennial grass matrix could fully establish under browsing and reach its highest total cover with similar values for all animal densities. Our simulation results were in line with findings of empirical (Archer et al., 2017; Knegt et al., 2008; Staver et al., 2009) and other modeling (Holdo et al., 2009; Langevelde et al., 2003) studies in similar savannas: Browsing pressure allowed a high cover of perennial grasses, which in combination with reduced resource availability could reduce shrub cover. Resource availability has been previously documented as a large determinant of shrub cover (Acharya et al., 2018).

Also, shrub cover was higher under high browsing intensities compared to lower browsing intensities. The shrub increase can be attributed to the greater dispersal of shrub seeds due to the larger number of herbivores (Archer et al., 2017), which we accounted for in our simulations.

### 4.2 Composition of strategy types

The response of the PFT community to the intensity and type of herbivory was strong, leading to significant shifts within the meta‐PFT composition. The simulation results showed that the variation of perennial grass strategy types between the land‐use scenarios was much stronger compared to that of shrub strategies, which showed a rather homogenous distribution of strategy types. The homogenous distribution of shrub strategy types might indicate that the pursuit of a particular strategy had a greater impact on perennial species than on shrubs. However, we were able to identify strategies for both meta‐PFTs that enabled them to dominate under specific herbivory pressure and to cope better with resource scarcity. We also detected a shift in dominance of specific strategies for an animal density between 30 and 40 ha/LSU, explained by the combined effect of herbivory pressure and shrub seed dispersal and establishment, which we discuss in the following.

#### 4.2.1 Composition of perennials grasses

Under low grazer and high browser density, the base type and strategies related to competitive water use (Cp), high drought resistance (Rp), as well as low palatability (Pr) were most successful in almost equal abundances. These strategies corresponded to species that were commonly found in the Namibian system studied here, for example, *Eragrostis echinochloidea* (Cp), *Stipagrostis uniplumis* (Rp), and *Bothriochloa radicans* (Pr).

In both browsing scenarios, woody and grass species coexisted and therefore competed for water (February et al., 2013; Tietjen et al., 2009). Not surprisingly, the strategies creating an advantage in dealing with the two main threats to the survival of grasses, herbivory and water limitation, were the ones that could prevail. When animal density was high, the trade‐off leading to high palatability became less important, as the plant biomass demand by animals usually exceeded available edible biomass and thus all edible biomass got completely removed, regardless of the palatability of a certain PFT. However, plants with a more efficient resource use could recover quicker from grazing events, as they could allocate acquired water directly into new plant growth (Abraham et al., 2019; Roodt, 2015).

In the scenario with low browser and grazer density, the strategy with low palatability and low drought resistance (Pr) became the most common one besides the base type. Under low grazing pressure, grazing happened very selectively on palatable strategy types. Also, as less shrubs were present, belowground competition for water was reduced to an extent, where most perennial grasses had access to sufficient water and could hence survive. Interestingly, the base type seemed to generally be well adapted to varying environmental conditions in our model. This was in accordance with our initial aim that the base type acts as a generalist that is not subject to specialization along specific trade‐off axes and can therefore thrive in various environments, but with clear disadvantages if the stress level requires better adaptations.

#### 4.2.2 Composition of shrubs

The results suggested that shrubs respond more strongly to changes in resource availability due to competition with perennial grasses than to browsing pressure or land‐use intensity, irrespective of grazing/browsing pressure. When browsing pressure was marginal (grazing scenarios), the distribution of shrub strategy types was very homogeneous. For both land‐use types, the strategy types with increased drought resistance (Rc, Rd) were clearly dominant in the community, but the strategies with high herbivory defense (Dc, Db) were also slightly more abundant. The increase of plant types with high defense mechanisms was in line with results of other studies: in regions with high animal densities, shrubs develop, for example, structural (spines) or chemical defenses (tannins) in response to browsing animals (Kiker & Scogings, 2019; Owen‐Smith, 1993).

While we reproduced the dominance of perennial grass strategy types in the model according to strategies used by common local species, the local shrub species corresponding to the dominant shrub strategy types are rather rarely found at our study site (e.g., *Acacia nebrownii* (Rc), *Grewia olukondae* (Rd)). Potential reasons for this are discussed in section *Implications of herbivory for plant diversity*.

In contrast to the perennial grass base type, the shrub base type performed the worst in every scenario, suggesting that the strategy types were better competitors irrespective of their trade‐offs.

With these results, we were able to answer how plant functional abundance and composition respond to different land‐use types and intensities (Question 1). The results have shown that herbivory type and intensity were a main contribution in shaping vegetation structure in semi‐arid African savannas. Grazing, especially at high intensity, has significantly reduced grass cover and confirmed our hypothesis (i) that bush encroachment would arise under these conditions. High (but common) SRs of grazers exceeded the capacity of savanna rangelands. Although the reduction of shrub cover in response to browsing was not as strong as the grass response to herbivory, browser‐dominated communities reduced some pressure on grasses and allowed a coexistence of both vegetation types, confirming our second hypothesis. While the magnitude of grazing impacts was directly related to grazer densities, browsing impacts were less dependent on browser densities, which is in line with other studies (Holdo et al., 2009; Maron & Crone, 2006; Staver et al., 2021). Therefore, management decisions, especially for browser‐dominated communities, should consider factors other than animal density, and implement a land‐use strategy based on thorough studies with respect to soil, habitat composition, grazing and browsing capacity, and physical limitations (Bothma & du Toit, 2016; Briske, 2017; Stafford Smith et al., 2007).

Nevertheless, our study suggested several ecological and economic benefits of reducing SRs of grazing animals, such as the resulting increase in perennial grass cover leading to higher fodder availability and the reduction of woody vegetation, which can reduce or avoid costly supplementary feeding and mechanical removal of shrubs. Furthermore, empirical studies observed that native herbivore communities are also changing, with native grazers gradually being replaced by mixed feeders (Staver et al., 2021).

Our results have also shown that herbivory intensity can be a further important factor shaping the composition of plant communities. Under high animal densities, some trait specializations, such as low or high palatability, become less important as herbivores feed on any plant they can to fulfill their biomass requirements. One consequence of this could be that undesirable plants benefit from herbivory disturbance. These plants, less desirable in terms of edibility, could then exploit the competitive advantage and replace palatable strategy types with less efficient recovery mechanisms. A common species in savanna systems taking advantage of high grazing intensities is *Tribulus zeyheri*, which is often found near waterholes where grazing intensity is particularly high (Van Rooyen et al., 1994).

In summary, the intensity of herbivory plays a critical role in determining vegetation abundance and composition and should therefore be determined with care.

### 4.3 Biodiversity

For both land‐use types, total species richness was highest under high animal densities. However, total species richness in the scenario with high cattle density was solely driven by woody species, as all shrubs apart from the base type survived and all perennial grasses vanished. Browser‐dominated land use could clearly increase survival of perennial grasses and therefore their richness, but also allowed some shrubs to prevail. In the scenario with low grazer density, shrubs and perennial grasses coexisted in almost identical numbers. Woody species richness always decreased to a lower level than grasses but for the scenario with high grazing intensity.

Hence, it is no surprise that evenness, which quantifies how equal a community is in terms of species numbers (Schwartz et al., 2000), is highest in the low grazer density scenario. Both browsing scenarios result in similar evenness values, with the high intensity scenario being slightly higher due to the higher number of shrubs in this scenario. Moreover, the high grazing intensity scenario, which resulted in the highest richness value, was the scenario with the lowest evenness. Such inverse pattern of evenness to richness with a species richness primarily driven by woody species is commonly found in studies linking land use to biodiversity patterns (Revermann et al., 2017; Rutherford & Powrie, 2013; Rutherford et al., 2012). For instance, in a similar *Colophospermum mopane* dominated savanna, heavy grazing did not affect species richness, but changed plant composition and decreased species evenness (Rutherford et al., 2012). Consequently, a high number of species that only comprises one functional group is not necessarily an indicator of high biodiversity.

#### 4.3.1 Implications of herbivory for plant diversity

The two factors explaining the distribution of PFTs showed a clear separation between perennial grasses and shrubs and a separation between the sensitivity of species to herbivory. The factor loadings suggested that in scenarios where herbivory was particularly intense, non‐palatable or species with high defense or recovery mechanisms were favored.

The clusters indicated that the distances between shrub strategy types were smaller compared to the distances between perennial grass strategy types, although shrub strategy types were more diverse in their traits.

Although shrub abundance differed between scenarios, there were little differences between shrub strategy types. The homogenous distribution of shrub strategy types across land‐use strategies could either mean that traits did not exert a strong effect on their abundance or that we did not capture certain key traits in our strategies that are related to resource use. Accordingly, this could explain why the dominant strategy types in the model do not correspond to the dominant local shrub species. Another reason for this could be that shrubs respond more strongly to climatic conditions than to herbivory, as previous results suggested (Lohmann et al., 2018). However, we did not test for different precipitation frequencies and intensities in this study.

FDis was higher in the two grazing scenarios where shrubs dominated. Previous studies also linked higher FDis with disturbance although less grass strategy types were viable under high herbivory pressure (Costa et al., 2017). An increase of FDis with disturbance could mean that a high trait dispersion is not necessarily beneficial per se for the ecosystem and that certain traits have a greater impact than others. Indeed, higher FDis did not coincide with higher vegetation cover. The mismatch of higher FDis with high vegetation cover could indicate that a completely even distribution of traits within one meta‐PFT might not be ideal, but rather a low level of FDis with grass‐like traits dominating. Generally, lower levels of FDis coincided with higher levels of total cover, suggesting that savannas with a dense grass matrix may only be able to support trait variability up to a certain level. This is in agreement with results of other studies linking community weighted means and functional diversity (Costa et al., 2017; Wen et al., 2019).

These results show the response of species richness and evenness to the contrasting land use scenarios (Question 2): Herbivory was a key process affecting richness and evenness, with high grazing intensity in particular having a negative effect on grass diversity. The model suggested that low grazer stocking densities (>30 ha/LSU) enable long‐term coexistence of trees and grasses. The results of a study comparing the response of plant diversity to the limitation of movement of both managed wildlife (with a high proportion of large browsers) and of grazing animals also found that high wildlife density reduces woody plant diversity and cover, while low grazer intensity allowed grasses and woody plants to coexist (Cassidy et al., 2013).

Unexpectedly, we found that browsers in all densities allowed for higher coexistence of plant species, which confirms hypothesis (iii). We conclude that biodiversity should not be evaluated by total species richness, but rather by focusing on the conservation value of particular species or functional traits. Therefore, we could only partly confirm our third hypothesis that browser‐dominated land use would positively influence biodiversity, especially if total richness was regarded. Moreover, functional diversity and ecosystem services did not co‐increase linearly, but it seemed that only some functional traits, that is drought resistance or palatability, improved the performance of a strategy type and allowed growth and survival under disturbance. This is confirmed by another study that investigated the trait distribution of a savanna community in a heavily grazed bush encroached savanna. The authors found little trait variability within this community, but noted that stress‐avoiding and stress tolerance through efficient resource conservation were clearly the dominating processes determining community composition (Geissler et al., 2019). Therefore, high trait diversity was not necessarily a direct indicator of a healthy ecosystem if certain traits were not present in the community.

### 4.4 Water use and availability

Land‐use type and intensity had a clear effect on total vegetation cover, which correlated strongly with water used by plants. We observed a distinct separation between the scenarios where T/ET increased with total cover. Previous studies linking T/ET to leaf area index (LAI) supported this result and also found vegetation growth properties to mainly determine water use in the absence of water stress (Kato et al., 2004; Ren et al., 2019). Although there was no difference in total ET between the scenarios, the higher vegetation cover increased the water used by plants, while reducing water loss by evaporation.

Under browsing, the reduction of evaporative loss due to higher vegetation cover promoted water use through transpiration and resulted in an increase in T/ET. Consequently, water was used more efficiently for plant growth instead of being lost through evaporation, which is supported by other studies considering vegetation and T/ET (Kato et al., 2004; Ren et al., 2019; Wei et al., 2017).

In contrast to plant water use, soil moisture was neither affected by land‐use type nor by intensity. The constant soil moisture level in this study indicated that it was not affected by vegetation cover and type and depended thus solely on precipitation. This can be explained by two aspects: First, the precipitation intensity of single rainfall events was rather low, leading to almost full infiltration of the rain into the rather sandy soil and to very low water losses by runoff, independent of vegetation cover. Second, potential evaporation exceeded the available water by far; therefore, losses by evapotranspiration were very fast, no matter if losses occurred mainly by evaporation or by transpiration.

Overall, we conclude that browser‐dominated when compared to grazer‐dominated land use could sustain a higher total vegetation cover given the same rainfall and soil moisture, answering our third question and confirming our fourth hypothesis that browser‐dominated communities can lead to an improved water uptake. With browsing animals, a higher perennial grass cover could be sustained, which had positive implications for plant water use, but also erosion control (Archer et al., 2017). More water was used by plants and less water was lost by evaporation, leading to a more efficient ecosystem water use. As water could be used more efficiently, these positive implications underline the benefits of browser‐dominated land use in the face of extended drought periods under climate change.

## 5 CONCLUSION

We found several ecological benefits of shifting land use from grazing‐ to browsing‐dominated herbivore herds, even for high densities. First, as the cover of woody species was less affected by herbivory compared to grass cover, land use dominated by browsers involved lower risks of degradation compared to grazers. Second, browsers could strongly decrease the pressure on grasses and therefore served as an organic and cost‐efficient way to counteract woody plant encroachment. Furthermore, we found increased biodiversity across major plant functional groups and an overall increase in water uptake by plants in scenarios with browsers. Therefore, browsers could provide a buffer against adverse effects of climate change, such as decreased soil moisture and loss of vegetation cover, also implying a lower erosion risk.

As we aimed for extremes of herbivory to narrow the possible range of responses, we only included browsing animals into this study, although wildlife consists of a range of species with different feeding behavior, including grazers, mixed feeders, and browsers. In addition, especially in Namibia, the importance of integrated wildlife systems where domesticated and native animals are kept together, such as in conservancies and nature reserves, is increasingly being recognized. In future work, the model could be expanded to incorporate (1) mixed feeders, (2) the effects of spatially and temporally varying animal density (e.g., due to seasonal migration), (3) nonconsumptive impacts (e.g., trampling, tree removal), and (4) the combined impacts of animals and wildfires.

In summary, we provided a new route to examine direct effects of herbivory by browser‐dominated compared to grazer‐dominated communities and its impact on selected ecosystem functions, which may assist rangeland managers to make sustainable decisions in the future.

## CONFLICT OF INTEREST

The authors have no conflict of interest to declare.

## AUTHOR CONTRIBUTIONS

**Katja Irob:** Conceptualization (lead); Data curation (lead); Formal analysis (lead); Investigation (lead); Methodology (equal); Validation (lead); Visualization (lead); Writing – original draft (lead); Writing – review & editing (lead). **Niels Blaum:** Conceptualization (supporting); Funding acquisition (lead); Methodology (supporting); Project administration (lead); Supervision (supporting); Writing – review & editing (supporting). **Selina Baldauf:** Formal analysis (supporting); Investigation (supporting); Validation (supporting); Visualization (supporting); Writing – review & editing (supporting). **Leon Kerger:** Data curation (supporting); Formal analysis (supporting); Investigation (supporting); Methodology (supporting); Visualization (supporting). **Angelina Kanduvarisa:** Data curation (supporting); Resources (supporting). **Ben Strohbach:** Data curation (supporting); Investigation (supporting); Methodology (supporting). **Dirk Lohmann:** Methodology (supporting); Writing – review & editing (supporting). **Britta Tietjen:** Conceptualization (lead); Funding acquisition (lead); Investigation (supporting); Methodology (supporting); Project administration (lead); Supervision (lead); Validation (supporting); Writing – review & editing (supporting).

### OPEN RESEARCH BADGES

This article has been awarded <Open Materials, Open Data> Badges. All materials and data are publicly accessible via the Open Science Framework at https://doi.org/10.6084/m9.figshare.16974892.v1.

## Supporting information

Appendix S1

Appendix S2

Appendix S3

Supplementary Material

## Data Availability

All R‐scripts and respective model output data needed to reproduce the methods, results, and figures of the manuscript *“Browsing herbivores improve the state and functioning of savannas*: *a model assessment of alternative land use strategies”* by Katja Irob, Niels Blaum, Selina Baldauf, Leon Kerger, Ben Strohbach, Angelina Kanduvarisa, Dirk Lohmann and Britta Tietjen can be found at “figshare”. Link to access the data repository: https://doi.org/10.6084/m9.figshare.16974892.v1.
