# Supplementary figures and images for: Browsing herbivores improve the state and functioning of savannas: A model assessment of alternative land‐use strategies
Source: Ecol Evol. 2022 Mar 18;12(3):e8715. doi: 10.1002/ece3.8715 (PMC8931791; doi:10.1002/ece3.8715)

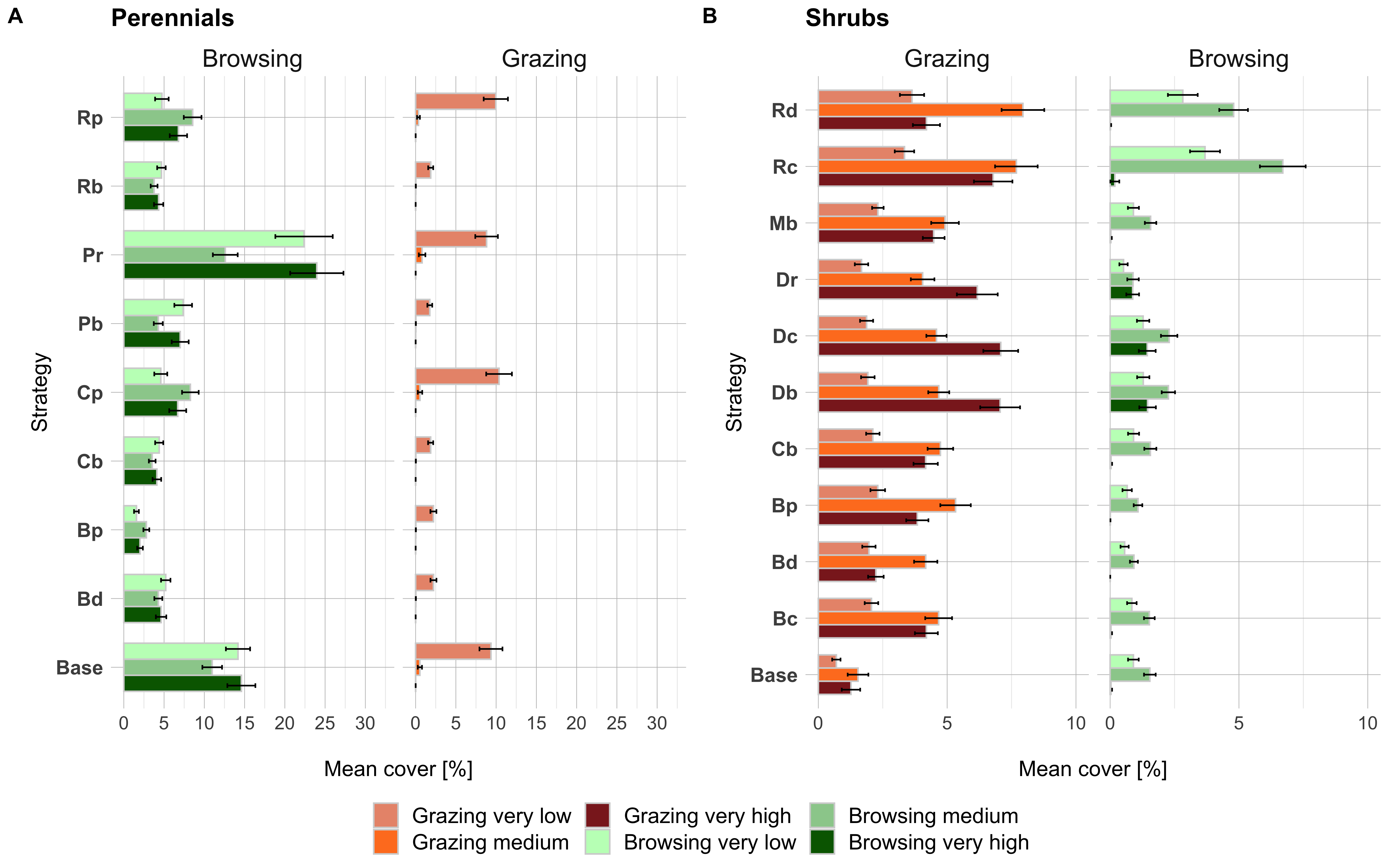

Supplement: Supplementary file 1 — Appendix S1 [file ECE3-12-e8715-s003.png]

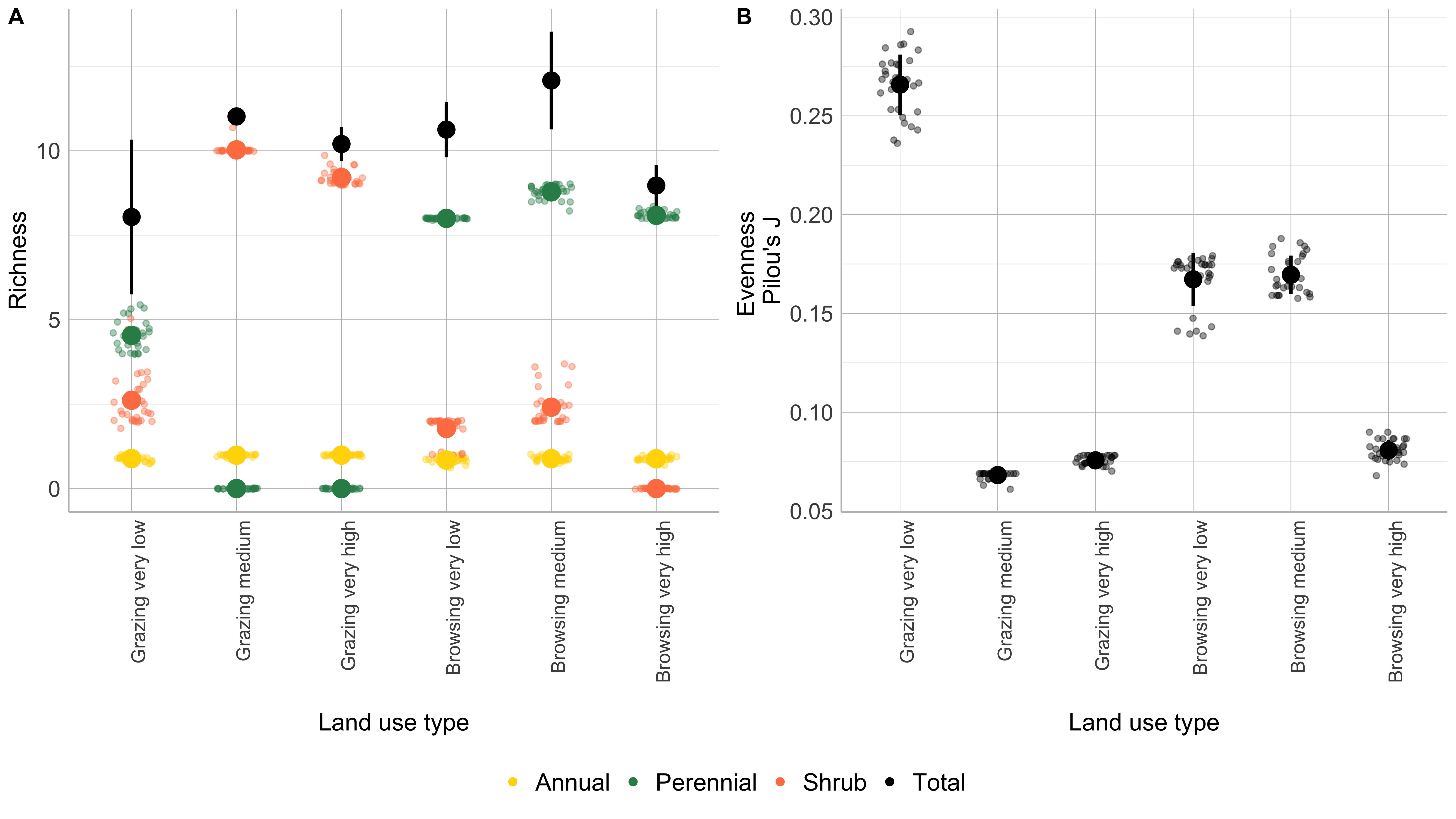

Supplement: Supplementary file 2 — Appendix S2 [file ECE3-12-e8715-s004.png]

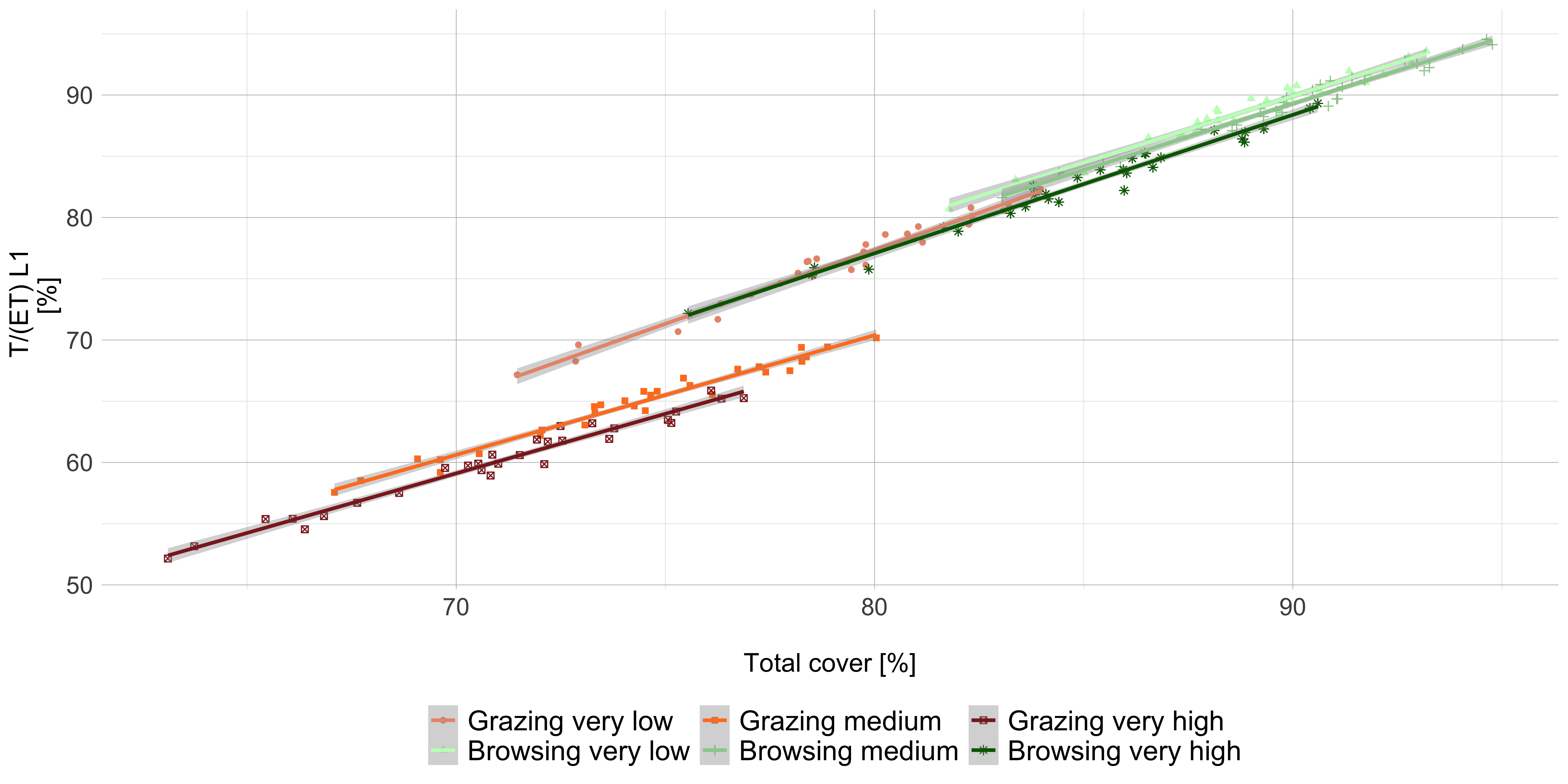

Supplement: Supplementary file 3 — Appendix S3 [file ECE3-12-e8715-s001.png]
