# Supplementary material for: Browsing herbivores improve the state and functioning of savannas: A model assessment of alternative land‐use strategies
Source: Ecol Evol. 2022 Mar 18;12(3):e8715. doi: 10.1002/ece3.8715 (PMC8931791; doi:10.1002/ece3.8715)
Supplement: Supplementary file 4 — Supplementary Material [file ECE3-12-e8715-s002.docx]

# Appendix

Supporting information

Irob, K. et al., Browsing herbivores improve the state and functioning of savannas: a model assessment of alternative land use strategies.

Submitted to *Ecology & Evolution*

**S1 Model rules**

The model we used for this study is based on the ecohydrological dryland model *EcoHyD* ([Tietjen et al., 2009](#_2bn6wsx); [Tietjen et al., 2010](#_3whwml4); [Lohmann et al., 2014](#_z337ya); [Guo et al., 2016](#_17dp8vu)). The model includes two sub-models: a hydrological and a vegetation sub-model (Fig. A.1). In the hydrological sub-model daily soil moisture in two layers (upper: 0-30 cm; lower: 30-80 cm) is calculated ([Tietjen et al., 2009](#_2bn6wsx)). The current version separates the actual evapotranspiration into soil evaporation and plant transpiration. Thus, we explicitly describe the process of evapotranspiration here while we do not describe other processes of the hydrological sub-model and a full description thereof can be found in Tietjen *et al.* (2009). The vegetation sub-model calculates biweekly growth of three plant functional types (PFTs), namely shrubs, perennial grasses and annual grasses ([Tietjen et al., 2010](#_3whwml4); [Lohmann et al., 2012](#_2jxsxqh); [Guo et al., 2016](#_17dp8vu)).


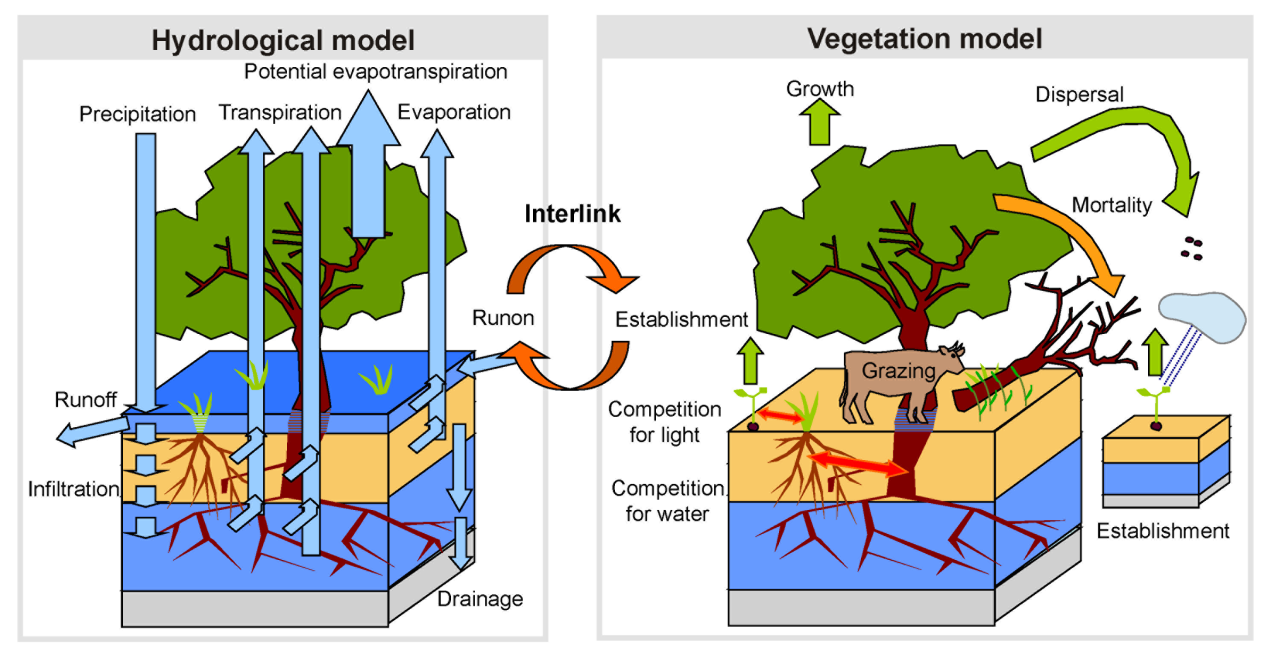


**Fig. S1.1** Overview of the hydrological and vegetation processes in the model EcoHyD, after Guo *et al.*  (2016)

Description, value and source of all model parameters are given for the hydrological (Table S.1.1) and the vegetation sub-model (Table S.1.2), respectively. Parameter and variable names directly relate to the description in Tietjen *et al.* (2009, 2010), Lohmann *et al.* (2012) and Guo *et al.* (2016).

**S1.1 Hydrological model**

*Evapotranspiration*

The assessment of potential evapotranspiration () follows the original description in Tietjen *et al.* (2009), in which is calculated based on the Hargreaves approach, which involves daily mean, minimal and maximal temperature $, and ) [℃]$, extraterrestrial radiation $()$, slope $(sl)$ and aspect effects $(af)$. The potential evapotranspiration function is (according to Tietjen et al., 2009):

$=0.0023*\left( \underline{T}+17.8 \right)***af*cos(sl)$ Eq. S1.1

Actual evapotranspiration is then calculated by applying the conceptual HBV model ([Hundecha and Bardossy, 2004](#_26in1rg)) to calculate the actual evapotranspiration from the surface (), the upper and the lower soil layer ($and$) using the volumetric soil moisture$) [/]$, stomata closing point, the cover of annual grasses, perennial grasses and shrubs ($, ,$) and the cover of each sub-PFT () (Tietjen, 2009). As in the version of Lohmann *et al.* (2012), annual grasses have only access to the upper soil layer, while perennial grasses and shrubs have access to water in both layers according to their root fraction (). The calculation functions of surface, upper and lower actual evapotranspiration are (according to Tietjen et al., 2009):

${ET}_{surf}= {ET}_{pot}*(1-0.5*\sum_{veg} {c\_total}_{veg}) [mm/d]$

${ET}_{L1}={ET}_{pot}*\left( \frac{W_{L1}}{wsc} \right)^{2}*g_{1}[mm/d]$

$$g_{1}=1.2-0.2*(\sum_{veg} {c\_total}_{veg}) [dimonsionless]$$

${ET}_{L2}={ET}_{pot}*\left( \frac{W_{L2}}{wsc} \right)^{2}*g_{2} [mm/d]$

with $g_{2}= \sum_{veg in perennial grasses, shrubs} {root}_{veg,L2}* [dimensionless]$

$=+ [mm/d]$ Eq. S1.2

Evapotranspiration was separated into evaporation and transpiration ([Guo et al., 2016](#_17dp8vu)), because plant dry matter production is directly linked to the water use, i.e. the transpiration of vegetation ([Miller et al., 2012](#_4i7ojhp)).

A model assumption is that evaporation $(E)$ is only relevant for surface water losses () and water losses from the upper soil layer (), while transpiration$(T)$ occurs in both soil layers. Therefore, actual evapotranspiration from the upper layer () is split into plant transpiration () and soil evaporation (). We established the relationship between the fraction of transpired water $(\frac{T_{L1}}{{ET}_{L1}})$ and total vegetation cover $(c\_total)$ in the upper soil layer ([Guo et al., 2016](#_17dp8vu)). In contrast, actual evapotranspiration from the lower layer () is completely converted into transpiration ().:

$$E=+$$

$$=$$

$$T=+$$

$$=+$$

$= \{1.05*c\_total-0.04 if >4\% 0 else$

$=$ Eq. S1.3

The next step is to determine how much water can be used by which plant functional type. This calculation did not change since the description of Tietjen *et al.* (2010) (Eq. S1.4). The relative uptake rate of each sub-PFT () is calculated based on the potential water uptake rate per biomass $()$ [mm * yr^-1^], vegetation cover $()$and the fraction of roots $()$ in the respective layer.

$= \frac{\theta_{veg} * {root}_{veg,Lx}}{\sum_{veg} \theta_{veg} * {root}_{veg,Lx} *c_{veg}} [dimensionless]$ Eq. S1.4

$T_{veg,Lx}=T_{Lx}*c_{veg}* [mm/day]$ Eq. S1.5

**S1.2 Vegetation model**

*Plant growth*

After the estimation of the fraction of transpired water in total evapotranspiration, we related the plant growth directly to transpiration instead of linking it to soil moisture as it was done in the original model version ([Tietjen et al., 2010](#_3whwml4)). In our model plant growth is implemented as an increase in the vegetation cover calculated in intervals of 14 days during a defined growing season. Growth of perennial grasses and shrubs (Eq. S1.6) is hereby based on a logistic behavior and calculated separately for each 5m x 5m grid cell. It depends on its own maximum cover (), its own current cover () and on the total cover of other vegetation formations () representing competition for space and light, meanwhile assuming a potential cover overlap between woody plants and grass (*lap*). The growth of annual grasses differs from the growth of perennial plants. Growth of annual plants (Eq. S1.7) does not include any competition for soil available water but exclusively depends on the size of empty space, potential growth rate () and general water availability in the upper soil layer (), as annual grasses are not assumed to invest resources in deep soil layers. However, the transpiration of annual grasses in the upper soil layer and its shading effect on evaporation from the surface are accounted for in the hydrological sub-model in the same way as is implemented for perennial grasses and shrubs. The growth functions for perennial grasses, woody plants and annual grasses are (according to Tietjen et al., 2010; Lohmann et al., 2012):

$=T_{veg}*r_{veg}*\left( 1-\frac{{c\_total}_{veg}}{{cmax}_{veg}-\left( {c\_total}_{\neg veg}*\left( 1- lap \right) \right)} \right)[]$ Eq. S1.6

${gr}_{ag}=min\left( 1*{avW}_{ag,L1}, 1 \right)*r_{ag}*\left( 1-{c\_total}_{pg}-{c\_total}_{s}*\left( 1-lap \right)-{c\_total}_{ag}-c_{mor} \right)[{yr}^{-1}]$

Eq. S1.7

*Plant mortality*

Two types of mortality affect vegetation cover. First, drought induced mortality () is calculated exactly as described in Tietjen *et al.* (2010). It is based on water availability and water uptake analogous to growth (see Eq. S1.4) and depends on a drought mortality rate , the average available water content in both soil layers during the growing season () and the relative water uptake rate ().

${md}_{veg,Lx}={mrd}_{veg}*c_{veg}*\left( 1-min\left( U_{veg,Lx}*{avW}_{veg,Lx},1 \right) \right)*\frac{{root}_{veg,Lx}}{\sum_{i} {root}_{veg,Li}} \left[ {yr}^{-1} \right]$

Eq. S1.8

Second, we introduced stochastic age-based mortality () for woody plants, referring to empirical data on *Acacia mellifera* L. from a semi-arid savanna similar to the one found in the study area ([Meyer et al., 2009](#_3j2qqm3)). This simulates a mortality that rather depends on the age of individuals than on water stress like for example infestation by fungi ([Joubert et al., 2008](#_35nkun2)). This senescence is applied to all cells with cohorts of shrubs older than the average age of death of individual trees ($ScenAge)$ ([Meyer et al., 2007](#_1y810tw)). The age of a cohort is determined by the date of the last establishment event that occurred in the respective cell. Hence, cells where the last establishment event of woody vegetation has been more than $ScenAge$ are completely cleared from woody vegetation with an annual probability $).$

*Plant biomass*

While vegetation cover of different sub-PFTs is the basic unit used in most equations (see above), plant biomass is necessary for some of the calculations, too. Hence, biomass is calculated based on vegetation cover and precipitation, the latter being the single-most important driver of biomass production from a given vegetation cover in semi-arid savanna ecosystems ([Snyman and Fouché, 1993](#_2xcytpi)). Specifically, biomass () is deduced from cover () and the average biomass produced per unit of cover given an average annual rainfall () depending on the following linear relation:

$=**cf(rain)$ Eq. S1.9

The slope of this relation is adapted by $cf(rain)$ to account for the dependence of biomass production on the precipitation in the current year ($rain$). The value of $cf\left( rain \right)$ is 1 in case of an average annual rainfall, below 1 in case of lower and above 1 in case of higher annual rainfall amounts. It is given by a linear relation with a constant ($\leq1$) determining the strength of precipitation influence on the cover-biomass relation ([Lohmann et al., 2012](#_2jxsxqh)).

$cf\left( rain \right)=rain*\frac{1-\beta_{r}}{MAP}+$ Eq. S1.10

*Herbivory*

Herbivory was described in the main part of the manuscript in the section *Grazing and browsing herbivory.*

*Dispersal and seedling establishment*

Dispersal and establishment are simulated as addition to the cover of a respective growth form () to certain cells in the grid. Germination and seedling/juvenile survival are therefore rather implicitly included (Tietjen *et al.* 2010).

Dispersal and establishment of perennial grasses was implemented as described by Tietjen *et al.* (2010). We assume no dispersal limitation on the given spatial scales ([Jeltsch et al., 1997](#_lnxbz9)), i.e. spatially homogeneous distribution of grass cover with the amount of cover depending on the mean perennial grass cover of the whole grid. Annuals are assumed to be always present as seeds and start off at every season without initial cover (i.e. no dispersal and establishment calculation, solely growth function determines occurrence). Woody plants are, in accordance with literature on regional typical shrub and tree species (i.e. *Acacia* species), assumed to be limited in dispersal, seed production and especially in establishment ([Meyer et al., 2007](#_1y810tw); [Joubert et al., 2008](#_35nkun2)).

However, the establishment of shrubs was simulated in more detail compared to the model version of Tietjen *et al.* (2010). Dominant encroacher species in semi-arid African savannas are known to have relatively high requirements regarding water availability for seed production, seedling germination and successful establishment. Different studies showed, that at least two subsequent years of above average rainfall are needed for successful establishment of *A. mellifera* ([Meyer et al., 2007](#_1y810tw); [Joubert et al., 2008](#_35nkun2)) and other woody plant species of semi-arid savannas ([Wilson and Witkowski, 1998](#_3as4poj)). Hence, successful establishment of woody plants is only possible if the mean soil-water content in the upper soil layer during the growing season is well above the wilting point of plants during two subsequent years$\left( W_{L1,mean}>{m_{est}*W}_{wp, s} \right)$. The factor was calibrated so that establishment conditions at one location occur on average 5-6 times per century ([Joubert et al., 2008](#_35nkun2)). To account for positive impacts of grazing on dispersal and establishment of woody plants ([Kraaij and Ward, 2006](#_1ksv4uv); [Hiernaux et al., 2009](#_3rdcrjn)), a grazing dependent factor was added to the function of Tietjen *et al.* (2010), so that amount and spatial extent of shrub establishment increases with increasing grazing pressure ([Ward and Esler, 2011](#_qsh70q)). This is achieved by linearly altering the parameters that determine the exponential decrease of “seedlings” (i.e. cover) with distance ($distConst$) and the maximum dispersal distance (${distmax}_{s}\left( SR \right)$).

The dispersal and establishment of shrub seedlings added as cover to a target cell ($ds$) is calculated for every source cell in the grid if the target cell had a sufficient water availability during the last and current growing season and its position was within the maximum dispersal distance according to the following term:

$ds=c_{s\_source}*{est}_{s}*{dist}_{0}*e^{-distConst\left( SR \right)*dist}*max\left( 1-{c\_total}_{s}-{c\_total}_{pg};0 \right) \left[ dimensionless \right]$

Eq. S1.11

Establishment and dispersal consequently depend on shrub cover in the source cell (), mean rate of seedling establishment (), cover of grasses and shrubs in the target cell ($,$) and the shape of an exponential dispersal decline (dependent on and $distConst$) as well as on the distance of the target from the source cell ($dist$). Grazing affects the dispersal kernel by the following linear relation being a function of the stocking rate ($SR$):

$distConst\left( SR \right)={dc}_{a}+\left( {dc}_{b}*SR \right) \left[ dimensionless \right]$ Eq. S1.12

The maximum dispersal distance ${distmax}_{s}\left( SR \right)$ is calculated so that the added cover $ds$ is at least 1% of the maximum possible value of $ds$ at the center of the source cell ($dist=0$).

**S1.3 References**

Guo, T., Lohmann, D., Ratzmann, G., Tietjen, B., 2016. Response of semi-arid savanna vegetation composition towards grazing along a precipitation gradient—The effect of including plant heterogeneity into an ecohydrological savanna model. *Ecol Model 325*, 47-56.doi:10.1016/j.ecolmodel.2016.01.004

Hiernaux, P., Diarra, L., Trichon, V., Mougin, E., Soumaguel, N., Baup, F., 2009. Woody plant population dynamics in response to climate changes from 1984 to 2006 in Sahel (Gourma, Mali). *J Hydrol 375*, 103-113.doi:10.1016/j.jhydrol.2009.01.043

Hundecha, Y., Bardossy, A., 2004. Modeling of the effect of land use changes on the runoff generation of a river basin through parameter regionalization of a watershed model. *J Hydrol 292*, 281-295.doi:10.1016/j.jhydrol.2004.01.002

Jeltsch, F., Milton, S.J., Dean, W.R.J., Van Rooyen, N., 1997. Analysing shrub encroachment in the Southern Kalahari: A grid-based modelling approach. *J Appl Ecol 34*, 1497-1508.doi:10.2307/2405265

Joubert, D.F., Rothauge, A., Smit, G.N., 2008. A conceptual model of vegetation dynamics in the semiarid Highland savanna of Namibia, with particular reference to bush thickening by Acacia mellifera. J Arid Environ 72, 2201-2210.doi:10.1016/j.jaridenv.2008.07.004

Kraaij, T., Ward, D., 2006. Effects of rain, nitrogen, fire and grazing on tree recruitment and early survival in bush-encroached savanna, South Africa. *Plant Ecol 186*, 235-246.doi:10.1007/s11258-006-9125-4

Lohmann, D., Guo, T., Tietjen, B., 2018. Zooming in on coarse plant functional types—simulated response of savanna vegetation composition in response to aridity and grazing. *Theor Ecol,11* doi:10.1007/s12080-017-0356-x

Lohmann, D., Tietjen, B., Blaum, N., Joubert, D.F., Jeltsch, F., 2012. Shifting thresholds and changing degradation patterns: climate change effects on the simulated long-term response of a semi-arid savanna to grazing. *J Appl Ecol 49*, 814-823.doi:10.1111/j.1365-2664.2012.02157.x

Lohmann, D., Tietjen, B., Blaum, N., Joubert, D.F., Jeltsch, F., 2014. Prescribed fire as a tool for managing shrub encroachment in semi-arid savanna rangelands. *J Arid Environ 107*, 49-56.doi:10.1016/j.jaridenv.2014.04.003

Meyer, K.M., Wiegand, K., Ward, D., 2009. Patch dynamics integrate mechanisms for savanna tree–grass coexistence. *Basic and Applied Ecology 10*, 491-499.doi:10.1016/j.baae.2008.12.003

Meyer, K.M., Wiegand, K., Ward, D., Moustakas, A., 2007. SATCHMO: A spatial simulation model of growth, competition, and mortality in cycling savanna patches. *Ecol Model 209*, 377-391.doi:10.1016/j.ecolmodel.2007.07.001

Miller, G.R., Cable, J.M., McDonald, A.K., Bond, B., Franz, T.E., Wang, L.X., Gou, S., Tyler, A.P., Zou, C.B., Scott, R.L., 2012. Understanding ecohydrological connectivity in savannas: a system dynamics modelling approach. *Ecohydrology 5*, 200-220.doi:10.1002/eco.245

Snyman, H.A., Fouché, H.J., 1993. Estimating seasonal herbage production of a semi‐arid grassland based on veld condition, rainfall, and evapotranspiration. *Afr J Range for Sci 10*, 21-24.doi:10.1080/10220119.1993.9638316

Tietjen, B., 2016. Same rainfall amount different vegetation —How environmental conditions and their interactions influence savanna dynamics. *Ecol Model 326*, 13-22.doi:10.1016/j.ecolmodel.2015.06.013

Tietjen, B., Jeltsch, F., Zehe, E., Classen, N., Groengroeft, A., Schiffers, K., Oldeland, J., 2010. Effects of climate change on the coupled dynamics of water and vegetation in drylands. *Ecohydrology 3*, 226-237.doi:10.1002/eco.70

Tietjen, B., Zehe, E., Jeltsch, F., 2009. Simulating plant water availability in dry lands under climate change: A generic model of two soil layers. *Water Resour Res 45*, 1-14.doi:10.1029/2007wr006589

Ward, D., Esler, K.J., 2011. What are the effects of substrate and grass removal on recruitment of Acacia mellifera seedlings in a semi-arid environment? *Plant Ecol 212*, 245-250.doi:10.1007/s11258-010-9818-6

Wilson, T.B., Witkowski, E.T.F., 1998. Water requirements for germination and early seedling establishment in four African savanna woody plant species. *J Arid Environ 38*, 541-550.doi:10.1006/jare.1998.0362

**S2 Model parameters**

**Table S1.1** Standard parameters and soil specific parameters in the vegetation sub-model (for further details see Tietjen et al., 2010; Lohmann et al., 2012; Guo et al., 2016).

| **Name** | **Description** | **Value** | **Unit** |
| --- | --- | --- | --- |
| $sclim$ | cover boundary for shrub (differentiate shrub and non-shrub) | 0.001 | - |
| $scrlim$ | cover boundary for shrub (differentiate juvenile and adult) | 0.1 | - |
| $lap$ | cover overlapping between grass and shrub | 0.2 | - |
| $bm\_c\_rain$ | constant for impact of precipitation on the biomass per unit of cover | 0.35 | - |
| $ga$ | constant for shaping quadratic function of grazing damage | 0.8 | - |
| $gb$ | constant for shaping quadratic function of grazing damage | 0.1 | - |
| $EnScov$ | cover boundary for shrub encroachment | 0.4 | - |
|  | relative uptake rate per perennial grass biomass | 0.9 |  |
|  | relative uptake rate per shrub biomass | 0.5 |  |
|  | relative uptake rate per annual grass biomass | 0.2 |  |
|  | fraction of roots in the upper layer for perennial grass | 0.63 | - |
|  | fraction of roots in the upper layer for shrub | 0.36 | - |
|  | potential growth rate of annual grass | 1.5 | $*$ |
|  | potential growth rate of perennial grass | 0.5 | $*$ |
|  | potential growth rate of shrub | 0.15 | $*$ |
|  | mortality rate dependent on soil moisture for perennial grass | 0.54 | $*$ |
|  | mortality rate dependent on soil moisture for shrub | 0.12 | $*$ |
|  | mortality rate dependent on soil moisture for annual grass | 0.8 |  |
|  | maximum cover for perennial grass | 1.0 | - |
|  | maximum cover for shrub | 0.8 | - |
|  | biomass at 100% cover for perennial grass | 1.9*10^6^ | $g*$ |
|  | biomass at 100% cover for shrub | 2.1*10^7^ | $g$ |
|  | biomass at 100% cover for annual grass | 1.7*10^6^ | $g*$ |
|  | non-edible biomass fraction for perennial grass | 0.15 | - |
|  | non-edible biomass fraction for shrub | 0.9 | - |
|  | non-edible biomass fraction for annual grass | 0.05 | - |
|  | grazing preference for perennial grass | 1 | - |
|  | grazing preference for shrub | 0.3 | - |
|  | grazing preference for annual grass | 0.6 | - |
|  | rate of successful establishment for perennial grasses | 0.05 |  |
|  | rate of successful establishment for shrub | 0.005 |  |
|  | factor determining  minimum mean soil moisture content for establishment for perennial grass | 1.05 | - |
|  | factor determining  minimum mean soil moisture content for establishment for shrub | 1.205 | - |
|  | constant for exponential dispersal decline with distance for shrub | 0.5 | - |
|  | constant for exponential dispersal decline with distance for shrub | 0.1 | - |
|  | constant for exponential dispersal decline with distance for shrub | 0.0125 | - |
|  | fraction of reserved biomass that cannot be grazed for perennial grass | 0.15 | - |
|  | fraction of alive biomass that is transformed into reserved biomass for perennial grass | 0.25 | - |
|  | fraction of reserved biomass that cannot be grazed for annual grass | 0.05 | - |
|  | fraction of alive biomass that is transformed into reserved biomass for annual grass | 0.1 | - |

Table S2.2: Hydrological parameters for the soil texture of loamy sand based on Tietjen et al (2009).

| **Name** | **Description** | **Value** | **Unit** | **Source** |
| --- | --- | --- | --- | --- |
| *S_r_* | Effective suction | 61.3 | mm | [Rawls et al., (1992)](https://www.zotero.org/google-docs/?broken=ktDU2e) |
| *K_S_* | Saturated hydraulic conductivity | 59.8 | mm h^-1^ | [Rawls et al., (1992)](https://www.zotero.org/google-docs/?broken=QJviqZ) |
| *WP* | Wilting point | 8.8 | Vol% | Calculated depending on rw |
| *fc* | Field capacity | 16.7 | Vol% | [Rawls et al., (1992)](https://www.zotero.org/google-docs/?broken=n51we2) |
| *sat* | Water content at saturation | 16.7 | Vol% | [Rawls et al., (1992)](https://www.zotero.org/google-docs/?broken=mvJM13) |
| *Ef* | Evaporation factor | 0.16 | - | Calibrated |
| *rw* | Residual water content | 7.5 | Vol% | Measured |
| *FL_1,2_* | Infiltration rate into lower soil layer | 0.1 | - | Calibrated |
| *maxFL_2_* | Maximum total infiltration into lower soil layer | 1.5 | mm h^-1^ | [Rawls et al., (1992)](https://www.zotero.org/google-docs/?broken=WXFnK6) |
| *diffConst* | Diffusion coefficient constant | 0 | - | Calibrated |
| *depL_1_* | Depth upper layer | 300 | mm | Measured |
| *depL_2_* | Depth lower layer | 600 | mm | Tietjen et al. 2010 |

##

## S3 Calibration


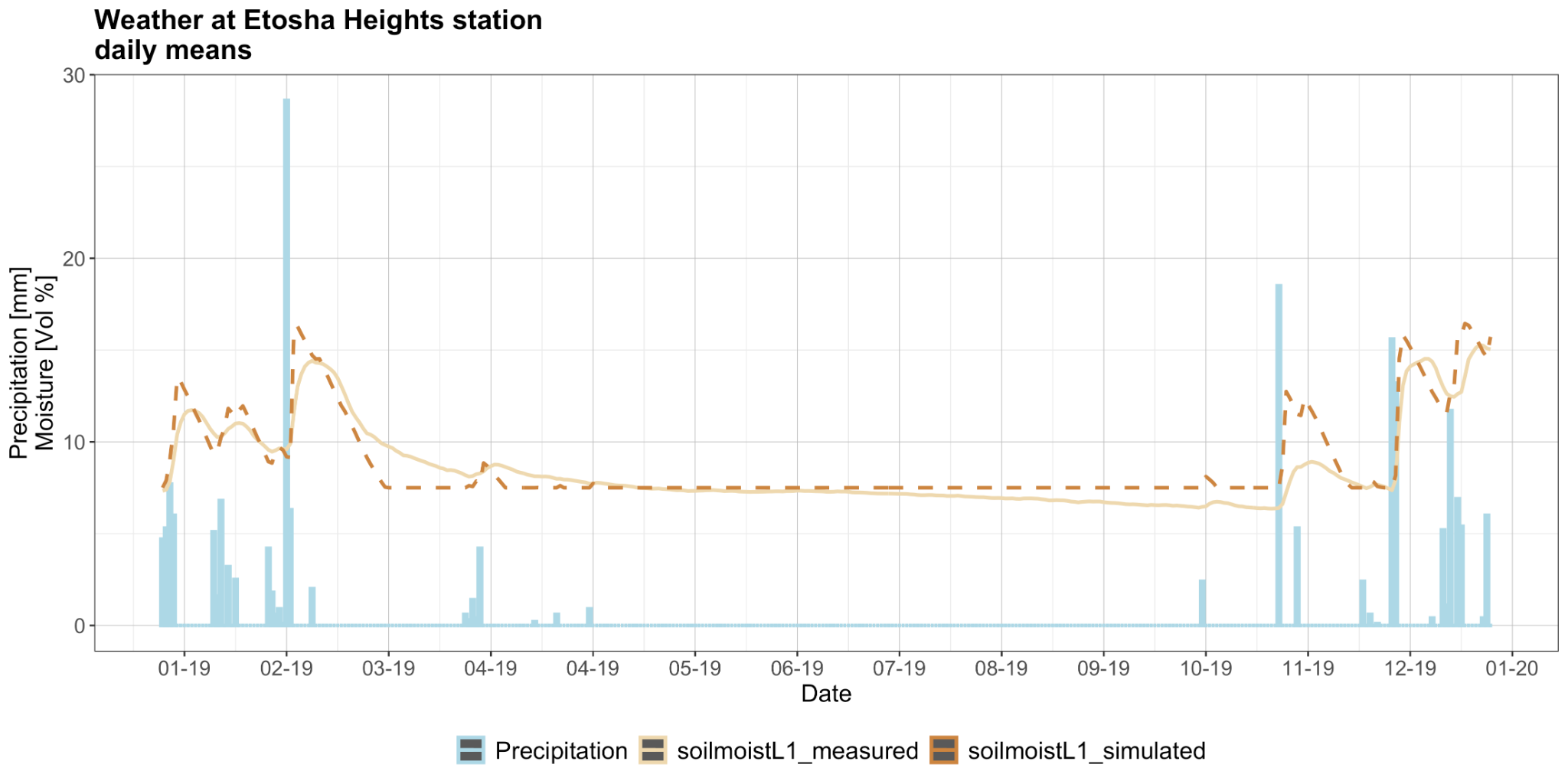


Figure S3.1: Measured (yellow) and simulated (orange/dashed) moisture of the first soil layer with loamy sand, forced by precipitation events in 2019 (RMSE = 1.092).

## S4 Sensitivity analysis

We performed a sensitivity analysis for perennial grass vegetation and shrubs to determine which parameter values resulted in a 10% cover increase or decrease relative to the mean cover of the base-type of the respective meta-PFT. We gradually varied the range of the standard parameter by 20-30% and determined by linear regression which parameter value would result in a 10% cover change. A detailed description can be found in the main part of the manuscript in the section *Model parameterization of plant species and sensitivity analysis.*


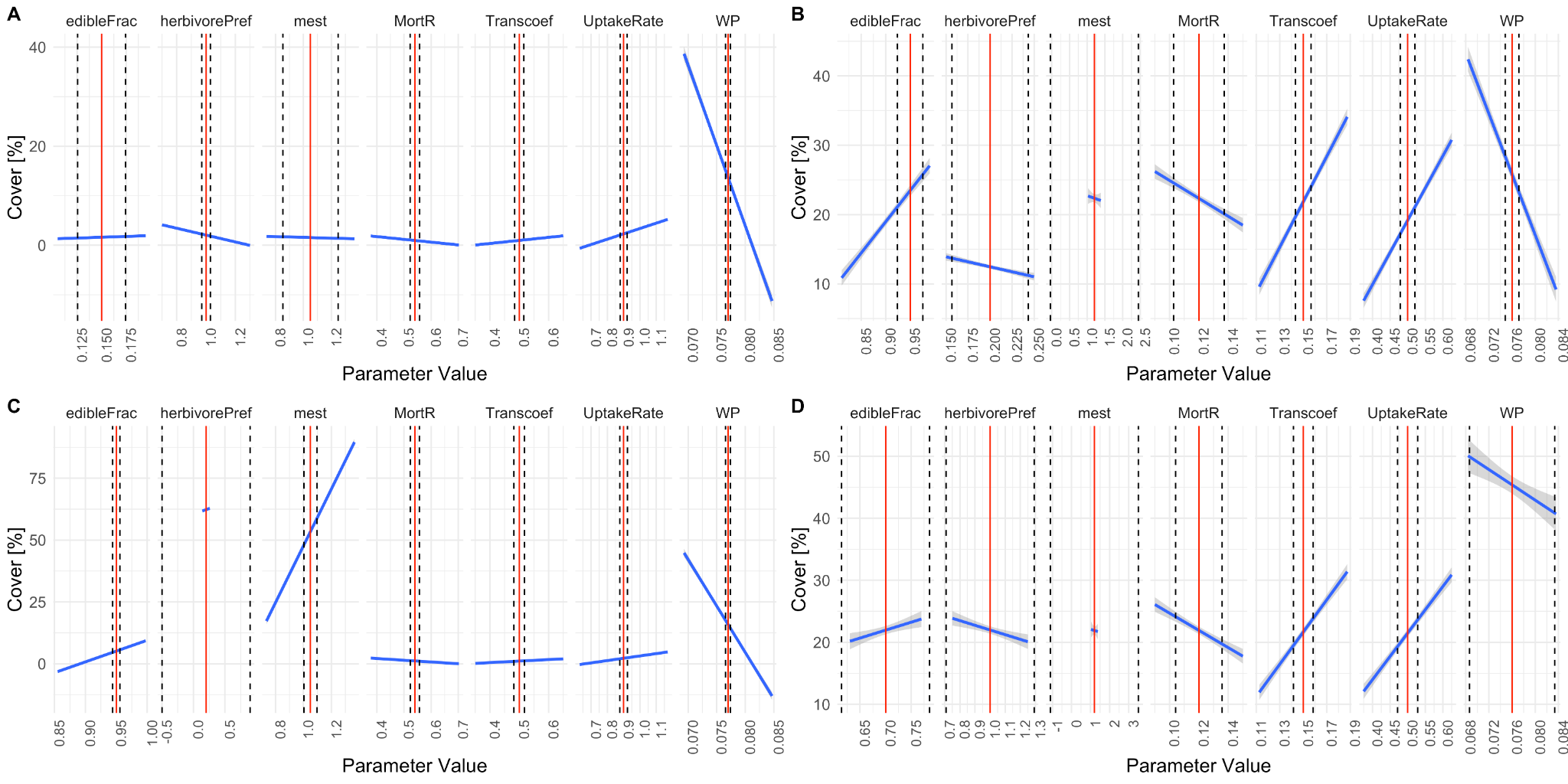


Figure S4.1: Results of sensitivity analysis for perennial grass parameters (left) under grazing (A) and browsing (C) and for shrub parameters (right) under grazing (B) and browsing (D).

## S5 Supplementary figures

Figure S5.1: Predicted mean cover ± SD [%] for 30 climate repetitions of the three PFTs for the whole simulation duration (lines) and the last 20 years of simulation (stacked bars) for all land use scenarios.

Figure S4.2: Mean cover ± SD [%] of perennial (*N = 9*) and shrub (*N = 11*) strategy-types in different grazing and browsing scenarios, suggesting which strategy-types dominate in a certain land use scenario. Results represent vegetation cover of the last 20 years of simulation repeated for 30 climate time series. Note that x-axes between perennial grasses and shrubs differ.

Figure S.3: Mean ± SD of species richness (A) and species evenness (B) under all land use scenarios for 30 climate repetitions. Every point represents one climate repetition. The left figure shows the total richness of all PFTs, as well as richness of each meta-PFT for every scenario respectively.

Figure S5.4: Correlation between the variation in the ratio of transpiration and evapotranspiration (T/ET) [%] as estimator for water use by plants and total plant cover [%] (b) separated for the different scenarios of land use. Results refer to water dynamics in the upper soil layer of the last 20 years of simulation and 30 climate repetitions.
